# Modelling the Impact of Artemisinin Combination Therapy and Long-Acting Treatments on Malaria Transmission Intensity

**DOI:** 10.1371/journal.pmed.0050226

**Published:** 2008-11-25

**Authors:** Lucy C Okell, Chris J Drakeley, Teun Bousema, Christopher J. M Whitty, Azra C Ghani

**Affiliations:** 1 Department of Infectious and Tropical Diseases, London School of Hygiene & Tropical Medicine, London, United Kingdom; 2 Department of Medical Microbiology, Radboud University Nijmegen Medical Centre, Nijmegen, The Netherlands; 3 MRC Centre for Outbreak Analysis and Modelling, Department of Infectious Disease Epidemiology, Imperial College London, London, United Kingdom; Hong Kong University, Hong Kong

## Abstract

**Background:**

Artemisinin derivatives used in recently introduced combination therapies (ACTs) for Plasmodium falciparum malaria significantly lower patient infectiousness and have the potential to reduce population-level transmission of the parasite. With the increased interest in malaria elimination, understanding the impact on transmission of ACT and other antimalarial drugs with different pharmacodynamics becomes a key issue. This study estimates the reduction in transmission that may be achieved by introducing different types of treatment for symptomatic P. falciparum malaria in endemic areas.

**Methods and Findings:**

We developed a mathematical model to predict the potential impact on transmission outcomes of introducing ACT as first-line treatment for uncomplicated malaria in six areas of varying transmission intensity in Tanzania. We also estimated the impact that could be achieved by antimalarials with different efficacy, prophylactic time, and gametocytocidal effects. Rates of treatment, asymptomatic infection, and symptomatic infection in the six study areas were estimated using the model together with data from a cross-sectional survey of 5,667 individuals conducted prior to policy change from sulfadoxine-pyrimethamine to ACT. The effects of ACT and other drug types on gametocytaemia and infectiousness to mosquitoes were independently estimated from clinical trial data. Predicted percentage reductions in prevalence of infection and incidence of clinical episodes achieved by ACT were highest in the areas with low initial transmission. A 53% reduction in prevalence of infection was seen if 100% of current treatment was switched to ACT in the area where baseline slide-prevalence of parasitaemia was lowest (3.7%), compared to an 11% reduction in the highest-transmission setting (baseline slide prevalence = 57.1%). Estimated percentage reductions in incidence of clinical episodes were similar. The absolute size of the public health impact, however, was greater in the highest-transmission area, with 54 clinical episodes per 100 persons per year averted compared to five per 100 persons per year in the lowest-transmission area. High coverage was important. Reducing presumptive treatment through improved diagnosis substantially reduced the number of treatment courses required per clinical episode averted in the lower-transmission settings although there was some loss of overall impact on transmission. An efficacious antimalarial regimen with no specific gametocytocidal properties but a long prophylactic time was estimated to be more effective at reducing transmission than a short-acting ACT in the highest-transmission setting.

**Conclusions:**

Our results suggest that ACTs have the potential for transmission reductions approaching those achieved by insecticide-treated nets in lower-transmission settings. ACT partner drugs and nonartemisinin regimens with longer prophylactic times could result in a larger impact in higher-transmission settings, although their long term benefit must be evaluated in relation to the risk of development of parasite resistance.

## Abstract 2

Lucy Okell and colleagues predict the impact on transmission outcomes of ACT as first-line treatment for uncomplicated malaria in six areas of varying transmission intensity in Tanzania.

## Editors' Summary

**Background.:**

Plasmodium falciparum, a mosquito-borne parasite that causes malaria, kills nearly one million people every year. When an infected mosquito bites a person, it injects a life stage of the parasite called sporozoites, which invade human liver cells where they initially develop. The liver cells then release merozoites (another life stage of the parasite). These invade red blood cells where they multiply before bursting out and infecting more red blood cells, which can cause fever and damage vital organs. Some merozoites develop into gametocytes, which infect mosquitos when they take a blood meal. In the mosquito, the gametocytes give rise to sporozoites, thus completing the parasite's life cycle. Because malaria parasites are now resistant to many antimalarial drugs, the preferred first-line treatment for P. falciparum malaria in most countries is artemisinin combination therapy (ACT). Artemisinin derivatives are fast-acting antimalarial agents that, unlike previous first-line treatments, reduce the number of gametocytes in patients' blood, making them less infectious to mosquitos, and therefore have more potential to reduce malaria transmission. These compounds are used in combination with another antimalarial drug to reduce the chances of P. falciparum becoming resistant to either drug.

**Why Was This Study Done?:**

Because malaria poses such a large global public-health burden, there is considerable national and international interest in eliminating it or at least minimizing its transmission. Malaria control agencies need to know how to choose between available types of ACT as well as other antimalarials so as to not only cure malaria illness but also prevent transmission as much as possible. The financial resources available to control malaria are limited, so for planning integrated transmission reduction programs it is important for policy makers to know what contribution their treatment policy could make in addition to other control strategies (for example, the provision of insecticide-treated bed nets to reduce mosquito bites) to reducing transmission. Furthermore, in areas with high levels of malaria, it is uncertain to what extent treatment can reduce transmission since many infected people are immune and do not suffer symptoms or seek health care, but continue to transmit to others. In this study, the researchers develop a mathematical model to predict the impact on malaria transmission of the introduction of ACT and alternative first-line treatments for malaria in six regions of Tanzania with different levels of malaria transmission.

**What Did the Researchers Do and Find?:**

The researchers developed a “deterministic compartmental” model of malaria transmission in human and mosquito populations and included numerous variables likely to affect malaria transmission (variables were based on data collected in Tanzania just before the introduction of ACT). They then used the model to estimate the impact on malaria transmission of introducing ACT or other antimalarial drugs with different properties. The model predicted that the percentage reduction in the prevalence of infection (the fraction of the population with malaria) and the incidence of infection (the number of new cases in the population per year) associated with a 100% switch to ACT would be greater in areas with low initial transmission rates than in areas with high transmission rates. For example, in the area with the lowest initial transmission rates, the model predicted that the prevalence of infection would drop by 53%, but in the area with the highest initial transmission rate, the drop would be only 11%. However, because more people get malaria in high-transmission areas, the total number of malaria illness episodes prevented would be ten times higher in the area with highest transmission than in the area with lowest transmission. The model also predicted that, in areas with high transmission, long-acting treatments which protect patients from reinfection would reduce transmission more effectively than some common currently used ACT regimens which are gametocyte-killing but short-acting. Treatments which were both long-acting and gametocyte-killing were predicted to have the biggest impact across all settings.

**What Do These Findings Mean?:**

As with all mathematical models, the accuracy of the predictions made by this model depend on the many assumptions incorporated into the model. In addition, because data from Tanzania were fed into the model, its predictions are to some extent specific to the area. Nevertheless the Tanzanian setting is typical of sub-Saharan malaria-affected areas, and the authors show that varying their assumptions and the data fed into the model within realistic ranges in most cases does not substantially change their overall conclusions. The findings in this study suggest that in low-transmission areas, provided ACT is widely used, ACT may reduce malaria transmission as effectively as the widespread use of insecticide-treated bed nets. The findings also suggest that the use of longer-acting regimens with or without artemisinin components might be a good way to reduce transmission in high-transmission areas, provided the development of parasite resistance can be avoided. More generally, these findings suggest that public-health officials need to take the properties of antimalarial drugs into account together with the levels of transmission in the area when designing policies in order to achieve the highest impact on malaria transmission.

**Additional Information.:**

Please access these Web sites via the online version of this summary at http://dx.doi.org/10.1371/journal.pmed.0050226.

- This study is further discussed in a *PLoS Medicine* Perspective by Maciej Boni and colleagues
- The MedlinePlus encyclopedia contains a page on malaria (in English and Spanish)
- Information is available from the World Health Organization on malaria (in several languages)
- The US Centers for Disease Control and Prevention provides information on malaria (in English and Spanish)
- Information is available from the Roll Back Malaria Partnership on its approach to the global control of malaria, on artemisinin-based combination therapies, and on malaria in Tanzania

## Introduction

Since 2000, artemisinin combination therapies (ACTs) have become widely adopted as first-line treatment policy for uncomplicated P. falciparum malaria in many endemic countries in response to parasite resistance that rendered previous first line treatments ineffective [1,2]. A secondary factor in the policy choice of ACT has been the proven ability of the artemisinin component to reduce patient gametocytaemia and infectiousness more than previous first-line treatments [3–6], which shows potential to translate into a reduction in overall transmission intensity as use of ACT is scaled up [7]. With the renewed interest in minimising transmission and moving toward malaria elimination [8], it is increasingly important to evaluate the ability of antimalarial treatments not only to cure disease, but also to reduce transmission. Understanding how pharmacological properties of ACT and other antimalarials affect transmission, as well as choice of delivery strategies, can help to maximise the impact of available resources.

Evidence for transmission or disease reductions following ACT deployment initially came from studies in South East Asia [9–12] and Southern Africa [13] which recorded a reduction in population P. falciparum prevalence or disease incidence after ACT became the main treatment for clinical malaria in the area. Since then, data from a number of settings including Zanzibar [14] and Rwanda have demonstrated a significant reduction in malaria cases following ACT introduction. While these studies are consistent in suggesting a benefit of ACT, they had observational, time-trend designs without control groups, and it is unclear how much of the effect in these settings was attributable to ACT and how much to other factors including simultaneous introduction of vector control measures [11,13], changes in climatic conditions [10], and change in diagnostic quality control. Furthermore, these observations come from relatively low transmission settings. In higher-transmission settings, the asymptomatic reservoir of infection may limit the potential for transmission reduction by treatment of symptomatic cases, because greater exposure increases immunity and the chance of an infection remaining asymptomatic [15,16].

A previous mathematical model of symptomatic case management predicted moderate to substantial impact of the nonartemisinin-based antimalarials sulfadoxine-pyrimethamine (SP) and amodiaquine (AQ) on age-prevalence patterns of infection and rates of severe disease and mortality under scenarios of 40% or 100% coverage [17]. Rates of uncomplicated clinical malaria episodes were reduced in low but not high transmission settings. Understanding to what extent ACTs with their additional gametocytocidal effects can reduce transmission in settings where partially effective treatments are already in place would aid malaria control agencies who currently must choose how to divide resources between drug purchases, drug delivery strategies, and other control interventions. The decision to maximise the coverage of the ACT class of drugs beyond the formal health care sector has substantial cost implications given their higher price [18], but it would be supported if ACT were likely to have a substantial effect on transmission. Choosing to invest in improved diagnostic techniques can save on overtreatment of fever cases with ACT; however, the importance of this widespread presumptive treatment for keeping transmission levels down needs to be investigated.

With increasing investment in research, the range of drugs in development for treatment of malaria is widening and a greater number are also becoming available in combination with artemisinins as ACT [19]. The stronger gametocytocidal activity of artemisinin derivatives compared to previous first-line antimalarials [20] has received the most attention in relation to potential transmission reductions by treatment [7]. However, two other main drug properties impact on transmission: prophylaxis and efficacy at clearing parasitaemia. Artemisinin derivatives have a very short prophylactic time in relation to other antimalarials, and although they remain highly efficacious in most areas of the world due to lack of parasite resistance, there are nonartemisinin antimalarial regimens that are of comparable efficacy in some regions [21]. Knowledge of the relative importance of these different drug properties in reducing transmission could help guide policy makers in choosing between ACTs with different partner drugs, and in the longer term a replacement first-line treatment, in the eventuality of parasite resistance or prohibitive cost of ACTs.

Here we develop a mathematical model to describe the impact of ACTs and other antimalarials on P. falciparum malaria transmission intensity. We use data from a survey prior to ACT introduction covering six different transmission settings in Tanzania typical of malaria-endemic Africa, in order to characterise rates of infection, symptomatic episodes, and antimalarial use. We then estimate potential transmission reduction following introduction of ACT as a first-line treatment and examine how the size of the reduction depends on coverage, use of diagnostic testing, and how it compares to the impact of alternative drugs with different pharmacodynamics. In this way we aim to elucidate potential goals for ACT policy implementation and inform future choices of first-line treatment.

## Methods

### Data

We use data from a cross-sectional malariometric survey of 5,667 residents of Tanzania undertaken during the rainy season (March–June) of 2002 prior to the introduction of ACT in treatment policy or their licensure and wider availability in the private market. Details of the methodology and results of this survey are described in detail elsewhere [22]. For our analysis, villages were grouped by region (Kilimanjaro or Tanga) and altitude (<600 m, 600-1200 m, >1200 m) which is a good proxy for transmission intensity with an estimated entomological inoculation rate ranging from less than one infectious bite per person per year at high altitudes, to about 100 infectious bites per person per year at low altitudes [23]. The data were stratified by age groups of 0–4 y, 5–14 y, and 15–45 y. Symptomatic malaria was defined by fever (37.5°C or above) and parasite density over age- and altitude-specific thresholds calculated previously using the same data [24] (age <5 y, altitude <600 m: 4,000 parasites/μl; age <5 y, altitude >600 m: 1,000 parasites/μl; age 5–15 y, altitude <600 m: 500 parasites/μl; age 5–15 y, altitude >600 m: 250 parasites/μl; age 15–45 y, all altitudes: 500 parasites/μl). A summary of these data is presented in Table 1 and in Table II in Text S1. Use of antimalarials in the last 14 d was self-reported. In two areas of the cross-sectional survey (North and South Pare), parasite resistance testing to the then first-line antimalarial SP was carried out [25]. We assumed that triple and double mutations in the *P. falciparum dhfr* and *dhfs* genes, respectively, would cause parasitological failure, i.e., parasites would not be fully cleared by SP treatment and the infection would persist, while the double-mutant *dhfr* would be sufficient to reduce the effective prophylactic time of SP [26]. We also assumed that similar resistance levels were present in all regions in the survey to all widely used nonartemisinin antimalarials at the time (Table 2).

**Table 1:**
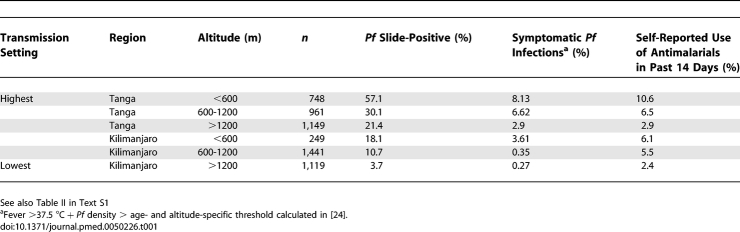
Summary of Data from Pre-ACT Cross-Sectional Surveys in Six Transmission Settings in Tanzania

**Table 2:**
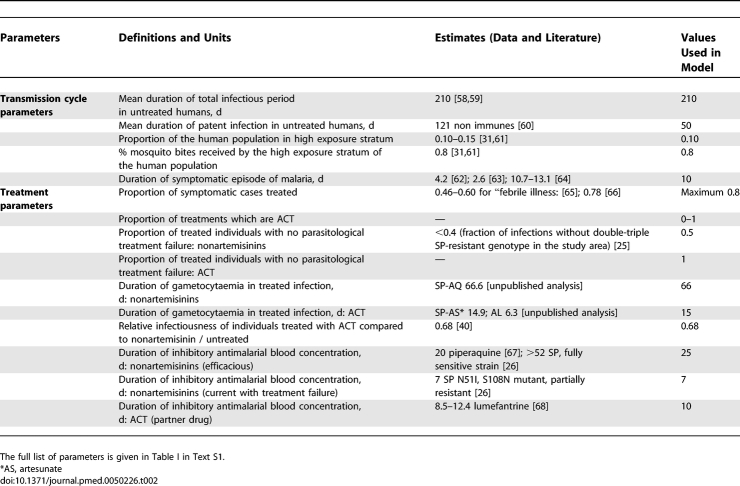
Key Parameters in the Model with Sources

Incidence rates of symptomatic malaria were calculated from the observed prevalence of symptomatic cases by region, altitude and age as prevalence/duration, using estimates of duration of symptoms from the literature (Table 2). Parasitaemia is assumed to remain asymptomatically once symptoms have ceased (see model below). We calculated age-specific treatment rates per person-year as the proportion reporting use of antimalarials / reporting time window (14 d) × 365. The relative infectiousness of different age groups was based on gametocyte densities (Tables I and II in Text S1).

### Mathematical Model

**Figure 1:**
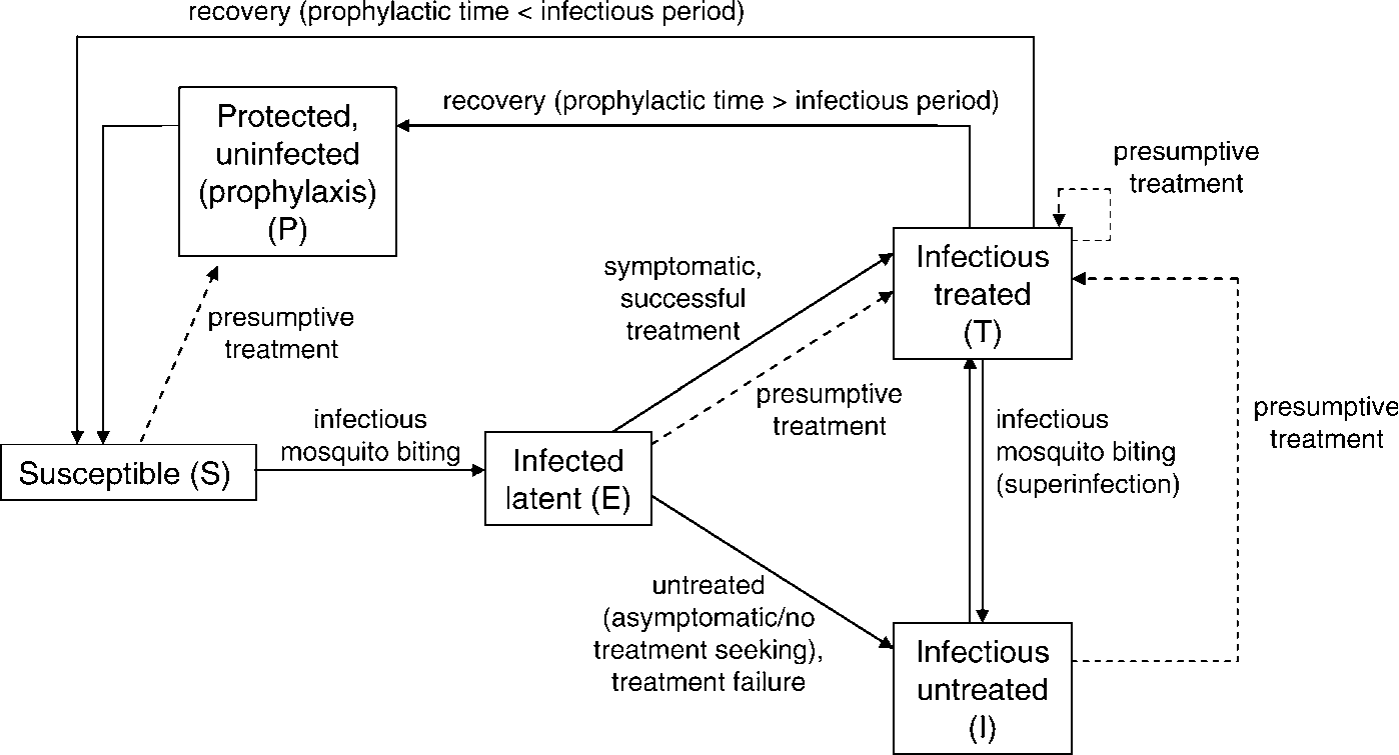
Overview of Model Structure The main states and transitions of the transmission cycle and treatment in the human population are shown, including presumptive treatment (dashed lines). Here one age and exposure group and a single type of antimalarial treatment are represented.

Malaria transmission is modelled in human and mosquito populations using a deterministic compartmental structure (Figure 1; Text S1). Humans are age-stratified and can be in one of five states: susceptible, *S*; latent, *E*; infectious and untreated, *I*; infectious and treated, *T*; or protected, *P*, where the latter state is included to model prophylaxis from treatment. Susceptible individuals become infected at a rate dependent on the density of mosquitoes, the human biting rate, the prevalence of infection in the mosquito, and the probability of developing blood stage infection following an infectious bite. The latent period is divided into two stages; during the first the parasites are liver- and early blood-stage and are subpatent (i.e., not detectable on a blood slide), and during the second stage the infection becomes patent. The infection then develops to the infectious, untreated state *I*, which is divided into four stages with infectiousness greatest in the first two stages and lower in subsequent stages to replicate patterns observed in longitudinal human-to-mosquito transmission experiments (Table I in Text S1) [27]. We assume that untreated infections are patent during the first three stages only [28]. Treated infections are described below. From the fourth stage of the infectious period, individuals recover to the susceptible state *S*. Superinfections (infection by a new parasite clone in addition to the original infection) occur independently of the initial infection at the same rate as in susceptible, uninfected individuals and upon superinfection individuals return to the first stage of the infectious period, extending the overall duration of parasitaemia. A proportion of infections and superinfections are symptomatic and we assume that symptoms occur at the beginning of an infection and are followed by a longer period of asymptomatic parasitaemia (Table 2) [29,30]. The model is stratified into three age groups (0–4 y, 5–14 y, and 15+ y) to correspond with the data. Malaria-attributable deaths are not explicitly incorporated. We allow for heterogeneity of exposure by additionally stratifying into two risk groups such that 10% of individuals receive 80% of bites from mosquitoes [31]. Immunity is modelled by incorporating age-dependency in the probability that an infectious bite develops into a blood stage infection [32], the proportion of infections which become symptomatic [33,34], the rate that an infection progresses to the final subpatent stage (but not the overall duration of infection [35]), and infectiousness to mosquitoes [36,37]. We assume negligible short-term change in immunity so that these parameters do not vary over time [38]. We also allow for an increase in exposure to mosquito bites with age due to increasing body surface area [32]. Susceptible vectors become infected at a rate dependent on the biting rate and the infectiousness of humans. They progress through latent and infectious stages but do not clear infection and die at a constant rate.

**Figure 2:**
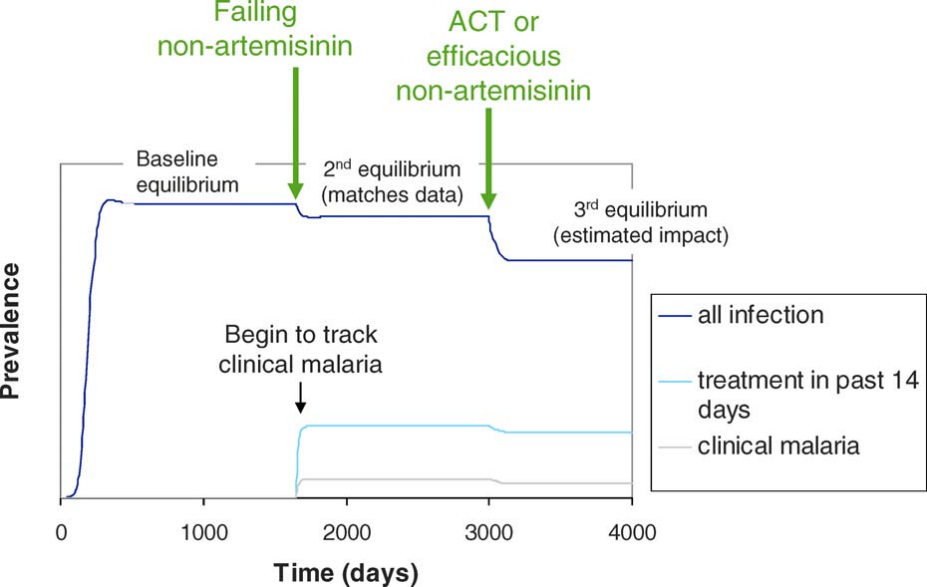
Example Model Run over Time for One Age and Exposure Group in One Survey Setting Initially this shows introduction of infection and baseline endemic equilibrium in the absence of treatment. Nonartemisinins with treatment failure are introduced first and the model is allowed to reach a second equilibrium, matching the prevalence of slide-positive malaria, clinical malaria and self-reported treatment in the Tanzania survey data. ACT or another antimalarial regimen are then introduced, and our main outcomes of interest are the reductions in prevalence and rate of clinical episodes between the second and third equilibriums.

Nonartemisinin treatment with a given rate of parasitological failure representing widely used antimalarials at the time of the Tanzania survey [25] is introduced after the model has reached endemic equilibrium in the absence of treatment (Figure 2). After reaching a second equilibrium in the presence of the nonartemisinin treatment, which represents transmission at the time of the survey in a given area, a proportion of the failing nonartemisinin treatments are replaced by ACT or another efficacious antimalarial and the model is allowed to reach a third equilibrium. A constant proportion of symptomatic individuals are treated. Treatment can reduce transmission in three ways in the model: by reducing the duration for which treated individuals are infectious (length of time gametocytes remain in the bloodstream), reducing their infectiousness (density and infectivity of gametocytes), and providing prophylaxis. The efficacy of an antimalarial also determines its impact on transmission. In our model, the infectious period of those in the treated state *T* is divided into four stages as in untreated individuals. The duration of the infectious period is shorter and the infection is assumed to become subpatent after the first stage. In the case of gametocytocidal antimalarials, infectiousness is reduced by a constant proportion throughout the infectious period relative to untreated individuals. The total duration of effective prophylaxis is equal to the duration of minimum inhibitory antimalarial concentrations in the blood minus the duration of liver stage infection, since current, widely used antimalarials protect only against blood stage parasites. We assume 100% protection from infection during this time. This includes protection from superinfection during the infectious period, and if prophylactic effects persist after parasites are cleared (depending on the antimalarial being used), individuals enter the protected state *P* before returning to the susceptible state *S*. Treatment clears infection with a certain percentage efficacy determined by the prevalence of infections consisting of parasites susceptible to the drug. Treated patients with resistant parasites experience parasitological treatment failure and enter the first stage of the untreated state *I*. Clinical failure, i.e. parasitological failure accompanied by symptoms, is not explicitly incorporated in the model. In our data from the Tanzania survey, individuals were asked if they had used antimalarials within the previous 14 d. Clinical failure usually occurs on average around 5 d after treatment [39], so we assume that re-treatment would occur relatively quickly after the first treatment episode. Therefore it would have little effect on the period prevalence measure of antimalarial use within the last 14 d, or the stage at which infection is treated. However, prevalence of symptomatic malaria was measured on the day of the survey only, so we use a relatively high estimate of duration of symptoms (Table 2) to allow for clinical failure. Individuals in any state can be presumptively treated for malaria regardless of parasitaemia, which provides a period of prophylaxis (see also Model Parameterisation and Validation, below). Infected individuals receiving presumptive treatment also move to the treated state. Full details of the model, parameter values, and justification of the model assumptions are given in Text S1.

### Model Parameterisation and Validation

The effects of nonartemisinin and ACT antimalarials on the infectiousness of patients to mosquitoes were estimated from antimalarial trial data, which measured human-to-mosquito transmission, taking into account subpatent gametocytaemia [3,40]. These data were also used to estimate the duration of infectiousness under different treatment regimens [unpublished analysis]. Patients were included in the trial regardless of pretreatment gametocytaemia, and therefore these estimates allow for lack of ACT effect on mature gametocytes [20]. Other parameter estimates were obtained from the literature. Key parameter values are given in Table 2 and a full list is provided in Table I in Text S1.

Following introduction of failing nonartemisinin treatment into the model, the product of the mosquito density and age-specific probability of mosquito-to-human transmission for each age group was varied so that at the subsequent second equilibrium the age prevalence of patent infection fitted the observed values for a given area in the survey data. The proportion of infections that developed symptoms was set for each age group so as to reproduce the observed symptomatic malaria age prevalence for a given area at the second equilibrium. These parameters, used in fitting prevalence in the model output at the second equilibrium, are known to have a wide range of possible values across different settings and age groups (Table I in Text S1) [32,41], therefore it was not necessary to set boundary values, and the model output matched the data exactly.

In the survey, an individual's infection status at the time of their reported treatment is unknown. Since presumptive treatment without testing for parasitaemia is common in the study area as in most malaria-endemic settings, it is likely that the reported treatment rate exceeds the rate of treatment of symptomatic malaria infections. We assumed that a maximum of 80% of incident symptomatic infections in each age group receive treatment (if the reported age-specific treatment rate in the population is sufficiently large; otherwise, this is calculated to match the prevalence of treatment history in the population). We assumed conservatively that any remaining treatment episodes were presumptive and were equally distributed within each age group regardless of infection state. The rate of presumptive treatment was calculated as the total age-specific rate of treatment per person-year in the population estimated directly from the data minus the age-specific rate of treatment of symptomatic cases (80% of the rate of symptomatic episodes per person-year estimated from prevalence of symptomatic malaria). We assumed that after introducing a new antimalarial there was negligible short-term change in presumptive treatment rates, but the rate of symptomatic malaria was allowed to change after introduction of a new treatment. We also explored the impact of introducing diagnostic testing (microscopy or antibody-based rapid diagnostic tests) together with ACT by removing presumptive treatment of those without a patent infection.

## Results

### ACT Impact on Transmission

**Figure 3:**
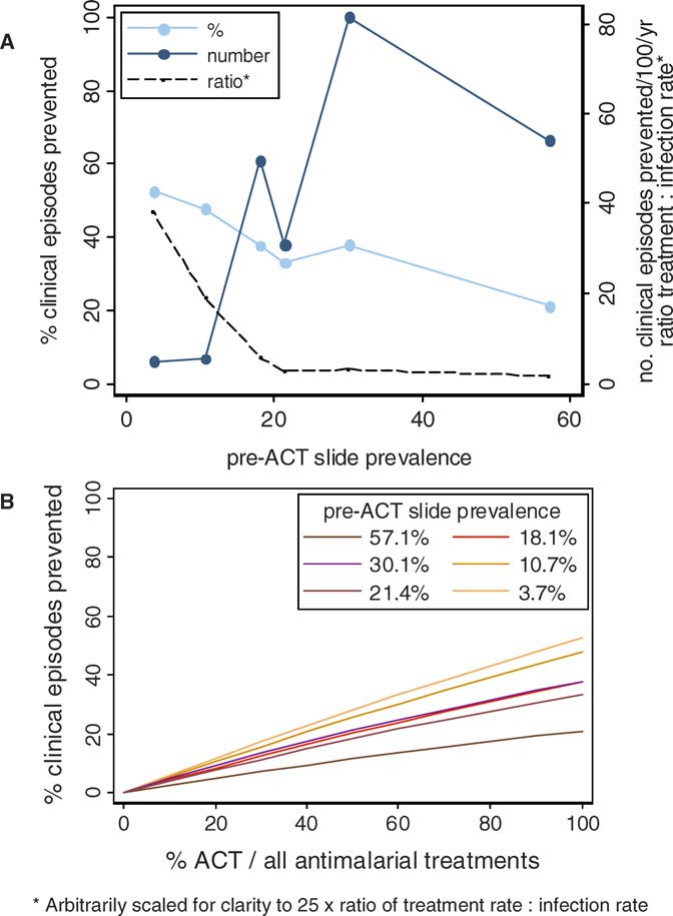
Model Predictions of ACT Impact on Transmission in Six Transmission Settings in Tanzania Compared to the Pre-ACT Scenario with Failing Nonartemisinin Treatment (A) Relative and absolute reductions in clinical episodes achieved by ACT if treatment rates remained the same and there was a 100% switch to ACT. Also shown is the pre-ACT ratio of treatment to infection rate. (B) Relative reductions in clinical episodes by ACT coverage.

The rates of infection, clinical episodes, and treatment in each transmission setting estimated from the survey prior to ACT introduction are shown in Table 3. The model-estimated impact of delivering ACT at complete coverage is shown in Figure 3A and Table 4. Replacing 100% of current antimalarial treatments with ACT without any change to the rates of antimalarial use in the study area is predicted to decrease the incidence of clinical episodes by between 21.1% and 52.5% and the prevalence of slide-positive infection by 11.5%–52.9%. This relative impact was largest in the setting with the lowest transmission levels pre-ACT (slide-prevalence = 3.7%), and there was a clear trend for decreasing impact as the pre-ACT transmission level became higher. This trend matched the ratio of the antimalarial treatment rate per person-year to the malaria infection rate per person-year in the populations, which also tended to decrease as pre-ACT transmission became higher (Figure 3A; Table 3). However, the absolute impact in terms of the numbers of clinical episodes averted was predicted to be highest in high transmission settings. An estimated 54.1 and 81.5 clinical episodes were prevented per 100 persons per year in the two areas of highest pre-ACT slide-prevalence compared to 4.9 clinical episodes per 100 persons per year in Kilimanjaro's lowest-prevalence area. In our analysis, reductions in clinical disease incidence are closely correlated with reductions in infection incidence, because short-term change in population immunity is assumed to be negligible, and therefore clinical disease is an indicator of transmission levels.

**Table 3:**
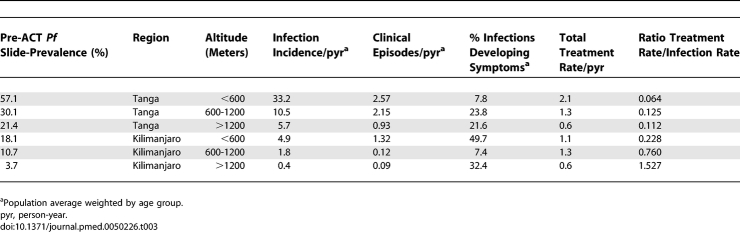
Estimates of Transmission, Disease, and Treatment Rates in Six Transmission Settings in Tanzania Prior to ACT from Survey Prevalence Data and the Model

**Table 4:**
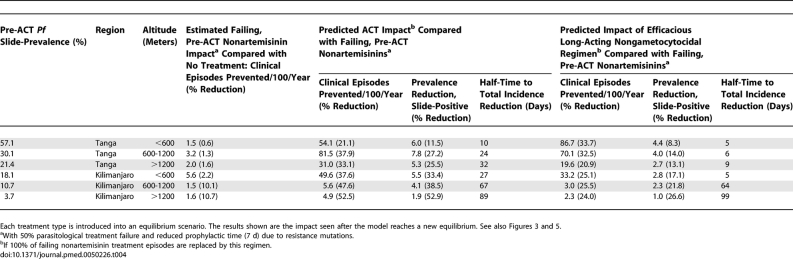
Model-Estimated Impact of Different First-Line Treatments on Transmission Outcomes in Six Transmission Settings in Tanzania

The time taken to achieve the reduction in infection incidence becomes longer as pre-ACT transmission intensity decreases (Table 4). With an instantaneous 100% coverage of ACT, 50% of the total reduction achieved is predicted to occur within 1–3 wk in the high-transmission settings, whilst it is predicted to take 2–3 mo in the lowest-transmission settings. The rate at which incidence decreases slows with time (Figure 2). Under scenarios of lower coverage of ACT where a proportion of individuals seeking treatment continued to be given the less effective, failing antimalarials used prior to ACT introduction, transmission reductions are predicted to decrease linearly with ACT coverage (Figure 3B). A full sensitivity analysis of our results is given in Text S1.

### Influence of Improved Diagnosis

**Figure 4:**
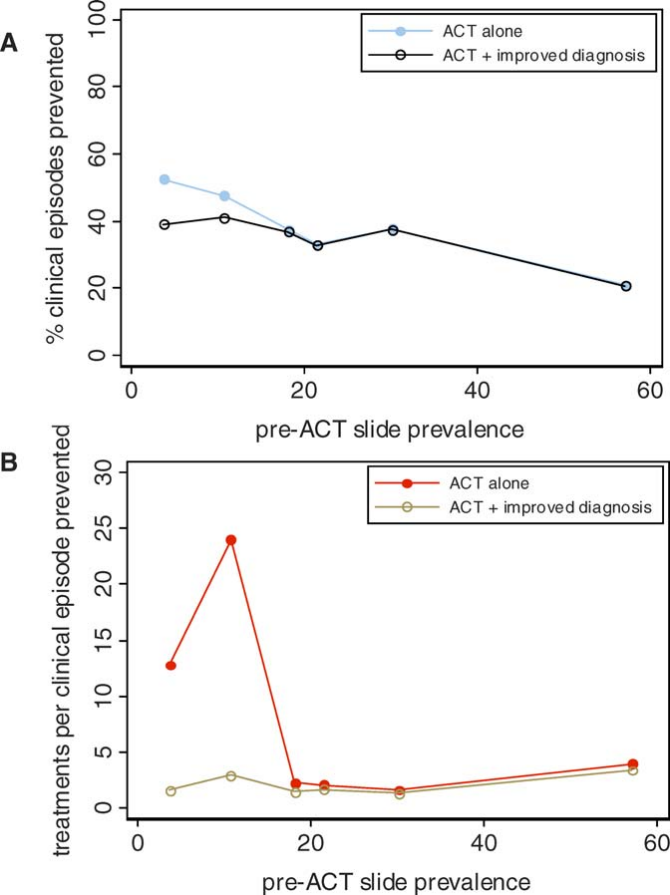
Model-Estimated Impact of Introducing Improved Diagnostic Procedures Improved diagnostic methods are introduced prior to antimalarial prescription together with a 100% switch to ACT in six transmission settings in Tanzania, compared to no change in current treatment use, (A) on the percentage of clinical episodes prevented and (B) on the efficiency of treatment at reducing transmission.

The importance of presumptive treatment to overall ACT impact was explored by modelling a scenario in which additional testing for the presence of parasites is incorporated into an ACT treatment programme. Under such a programme, individuals without detectable infection who seek treatment are no longer prescribed antimalarials; however, those with asymptomatic infection who have patent parasitaemia (for example, those who seek treatment due to symptoms from another illness) continue to be treated. In the two lowest-transmission settings in Tanzania, reported rates of treatment per person-year in the cross-sectional survey were higher than the estimated rates of clinical episodes (Table 3), suggesting greater overuse of antimalarials in these areas. Reducing treatment rates through introducing diagnosis reduced ACT impact to some extent in the two lowest transmission settings only (Figure 4A). In these settings, the model estimates that a relatively high proportion of antimalarial treatments are used by individuals without symptomatic malaria or detectable infection. This use of treatment reduces transmission less efficiently because it does not target the most infectious individuals, but has a small effect through cure of subpatent infections and prophylaxis. Similarly, the number of treatment courses required to indirectly prevent one clinical episode via transmission impact was greatly reduced by laboratory diagnosis in the two lowest-transmission settings only (Figure 4B). Improved diagnosis can reduce treatment rates and thus ACT impact further if we assume a lower proportion of treatment is used by symptomatic cases (Figure VIII in Text S1).

**Figure 5:**
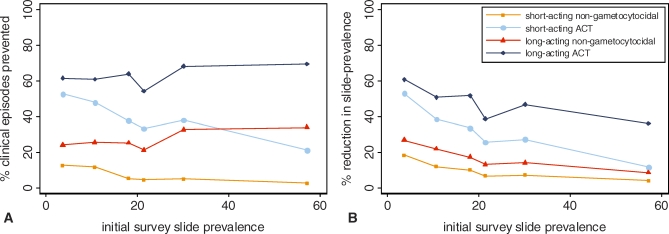
Model-Estimated Impact of Introducing Antimalarials with 100% Efficacy and Different Pharmacodynamic Properties Impact is shown on (A) clinical episodes and (B) slide-prevalence of infection in six transmission settings in Tanzania, compared to the pre-ACT scenario with failing nonartemisinin treatment assuming a 100% switch to these treatments. Short-acting nongametocytocidal: prophylactic time = 10 d, no specific gametocytocidal action. Short-acting ACT (as Figure 3A): prophylactic time = 10 d, with the gametocytocidal action of artemisinin. Long-acting nongametocytocidal: prophylactic time = 25 d, no specific gametocytocidal action. Long-acting ACT: prophylactic time = 25 d, with the gametocytocidal action of artemisinin.

### Impact on Transmission of Antimalarials with Different Pharmacodynamic Properties

In addition to investigating ACT impact on transmission, and thus the impact of a gametocytocidal drug, we used the model to examine the importance of the efficacy and prophylactic effects of antimalarials in reducing transmission (Figure 5). We consider the impact of individual antimalarials in order to separate out the effects of these drug properties, however this is intended to be purely theoretical since use of any antimalarial alone increases the chance of development of resistance [42]. Using data on SP, it is assumed that the nonartemisinin treatments in use in the study area at the time of the survey had only 50% efficacy and a prophylactic time of 7 d due to high levels of resistance mutations [25,26]. Our results suggest that these treatments were having little impact on transmission with an estimated 0.6%–10.7% lower rate of clinical episodes compared to a scenario without any antimalarials (Table 4). As before, the impact was largest in the lower-transmission settings.

Replacing 100% of the failing treatments with a theoretical, 100% efficacious nongametocytocidal antimalarial with a prophylactic time of 10 d (as assumed for a short-acting ACT) is estimated to reduce the rate of clinical episodes by 2.5%–12.6% (Figure 5A). Again this impact increased as pre-ACT transmission decreased. However, using a nongametocytocidal antimalarial that had a longer prophylactic time of 25 d as well as 100% efficacy substantially increased its impact on transmission (Figure 5). Clinical episodes were reduced by 24.0%–33.7% and, in contrast to the gametocytocidal drugs, this impact increased as baseline transmission increased. This relationship was observed despite the lower ratio of treatment rate to infection rate at higher transmission (see Figure 3A). In the setting with highest initial slide-prevalence (57.1%), the impact of such a drug on clinical episodes was 60% higher than the predicted impact of a drug with the gametocytocidal effects of ACT and a prophylactic time of 10 d (such as artemether-lumefantrine [AL], the most commonly used ACT) (Figure 5A; Table 4). An ACT with a long-acting partner drug (25 d prophylaxis) had the highest impact of all drug types, reducing rates of clinical episodes by 54.1%–69.1%, with the impact tending to increase with pre-ACT transmission levels (Figure 5A).

Gametocytocidal drug action was less effective in reducing transmission from an initially high than from a low level because following clearance of gametocytes individuals were more quickly reinfected. By contrast, prophylactic effects were more important for reducing new inoculations and clinical episodes in high- than in low-transmission settings, because there was a greater chance of receiving an infectious challenge during the prophylactic period. Prophylactic and, to some extent, gametocytocidal drugs are predicted to have less effect on infection prevalence than on the incidence of clinical episodes in higher-transmission settings (Figure 5B). A similar pattern is observed with other malaria control interventions [43] because of the nonlinear relationship between prevalence and incidence of malaria infection [44]. This relationship arises because the time period between new parasite inoculations is shorter at higher transmission and superinfection is more common. Therefore, preventing an inoculation does not reduce the amount of person-time spent infected, and therefore prevalence, as much as it would in a lower-transmission setting.

### Sensitivity Analysis

Model results were relatively robust to variations in the majority of key parameters (full details provided in Text S1), but we found that ACT impact size would be lower in populations with high heterogeneity in exposure (Figure V in Text S1). The size of ACT gametocytocidal effects in treated individuals as well as prophylactic effects was an important determinant of transmission reductions (Figure VI in Text S1). We also confirmed that the trends shown in the results still existed in a much simplified version of the model, which did not use the survey data and ignored presumptive treatment, treatment failure, age, and immune effects, assuming a constant proportion of infections treated in all areas. Gametocytocidal drugs still had most impact in lower-transmission settings, and the reverse was true for prophylactic drug effects (Figure IV in Text S1).

## Discussion

Our model shows the potential for an appreciable impact of ACT on malaria transmission at current rates of antimalarial treatment in our study area of Tanzania. The predicted reductions in prevalence of slide-positive infection at 100% coverage are between 11.5% and 52.9%, which compare with 13%–42% achieved by insecticide-treated bed nets (ITNs) in trial settings [43] and, as such, ACT could form an important part of a transmission reduction programme. The estimated reductions in rates of clinical episodes of 21.1%–52.5% with a short-acting ACT are lower than pooled estimates of ITN impact of 50%–62% [43]. In higher-transmission settings (>20% baseline slide-prevalence), ACT is predicted to have its smallest relative impact on transmission as found in a previous model looking at SP-AQ [17], due to a combination of a lower proportion of infections being treated and the different dynamics of infection prevalence (Figure 3A; Figure IV in Text S1). However, in terms of public health impact, the absolute number of clinical episodes prevented by introducing ACT in high-transmission settings was much greater, and the courses of treatment required per episode indirectly averted was substantially lower given current treatment rates across settings. Furthermore, the direct benefits of effective clearance of parasites for infected individuals would be considerable. More widespread use of diagnostic tools prior to treatment is predicted to increase the efficiency of ACT in reducing transmission per treatment course, especially in lower-transmission settings, although it could result in some reduction in the total impact, as previously suggested [45]. Depending on how quickly ACTs are made widely available, significant impact could be seen within a few weeks in higher-transmission settings but would occur over several months in lower-transmission settings. Our analysis deliberately represents an ideal scenario in order to estimate the maximum potential for ACT impact, whilst in reality the difficulties of imperfect patient adherence and of achieving good coverage in all health sectors [1,46] would reduce the speed and magnitude of transmission reductions, potentially substantially, as seen with ITNs outside trial settings.

Our analysis finds that gametocytocidal properties of antimalarials have the most relative impact in lower-transmission settings, while prophylactic effects have more impact in areas with high transmission. An ACT with the gametocytocidal effects of the artemisinin derivative and the prophylactic effect of a longer-acting partner drug is predicted to have the greatest impact on transmission across all areas in the short-term time scale of our model. ACT with long-acting partner drugs are currently in use in some areas; for example, artesunate-mefloquine is recommended by WHO. The more recently formulated dihydroartemisinin-piperaquine is also likely to become used more widely [47]. If used widely in the longer term in higher-transmission settings, the advantage of the prophylactic effects of these long-acting ACTs would need to be weighed against a higher selective pressure for resistant parasite strains [26,48,49]. Careful resistance surveillance would be important. Our finding that a long-acting nongametocytocidal drug regimen could have as much or more effect on transmission than a short-acting ACT in higher-transmission areas similarly needs to be considered bearing resistance development in mind. Such a drug regimen would need to contain two or more efficacious antimalarials in combination. Existing nonartemisinin combinations include SP-AQ and SP-chloroquine, but their use is limited due to widespread resistance [47]. However, the predicted impact of long-acting treatments has implications for future development of nonartemisinin combinations and their use as a first-line treatment, particularly if cost or development of artemisinin resistance make ACT a less attractive option. Developing and deploying drugs with stronger gametocytocidal activity than artemisinins would also be highly beneficial for transmission reductions according to our results (Figure VI in Text S1). Primaquine may be one such existing drug, but limited evidence does not show a consistent advantage over artemisinin derivatives [50,51].

Estimated treatment impact did not vary evenly with baseline slide-prevalence of infection, particularly in the medium-transmission settings (Figures 3A, 4A, and 5). This is likely to be due partly to sampling variation resulting from the smaller sample size in the area with 18.1% baseline prevalence (Table 1). The prevalence of symptomatic malaria and reported use of antimalarials are high in this area relative to the others given its measured infection prevalence, resulting in higher estimates of treatment impact. However, as has been found in other endemic populations [52], reported use of antimalarials also did not decline evenly with baseline prevalence in the other areas despite larger sample sizes. Variation in ACT impact due to treatment-seeking behaviour of populations is likely to be a real feature of ACT scale-up. Our results should be seen in terms of short-term impact only, since we do not take into account changes in the immunity of populations over time. The necessary data for our model, such as the incidence of infection and the proportion of infections developing symptoms, are rarely measured directly due to the difficulties of detecting all superinfections; therefore, we relied on model estimates from prevalence data, which gave results that were mostly within the range of the few data available [33,34]. Varying parameters and the model assumptions that affected these estimates caused limited change in the results except in the case of heterogeneity of exposure (see the sensitivity analysis in Text S1). In the highest-transmission setting the estimated incidence of infection and clinical episodes in the pre-ACT setting appear high (Table 3), but may be reasonable given measured entomological inoculation rates [23], and that the survey was conducted during the season of highest transmission and thus true annual rates would be lower. Whilst the prevalence of symptomatic malaria in the survey data decreased with transmission setting, our estimated proportion of infections developing symptoms did not show a clear trend of increasing as would be expected due to decreasing immunity (Table 3). This trend was visible across the four higher-transmission settings, but not in the two sites with lowest transmission intensity, probably due to the small numbers of cases meeting our definition of symptomatic malaria in these areas (Table 1 in the main text and Table II in Text S1). However, we had estimates of treatment intake in the population, and our results are relatively robust to whether this treatment occurs in symptomatic or asymptomatic individuals (Figure VIIa–b in Text S1). They are more sensitive to the accuracy of our treatment data, which is self-reported, and thus potentially underestimated [53]. Our data are limited to individuals under 45 y of age, which in these settings represents around 87% of the population [54]. By assuming in our model that individuals over 45 y have the same infection and immunity status as 15- to 45-y-olds, we may underestimate overall immunity levels in this age group to some extent. However, our sensitivity analysis showed that results were not affected substantially by ignoring age structure (Figure VIIi–j in Text S1). In generalizing our results to other settings it should be borne in mind that our slide-prevalence data represent the peak in annual transmission in these areas, whilst immunity levels in the study populations reflect lower transmission during other times of the year.

Our model predictions are roughly compatible with some of the trends from observational studies in areas where ACT was introduced, although it is uncertain to what extent these trends were attributable to ACT. In studies where transmission was similar to our lowest transmission setting (Kilimanjaro, >1,200 m) or lower, around 50% [10,12] to 75% [13] reductions in clinical episodes or prevalence occurred following ACT introduction, although there were also changes in vector levels during the same time period. In an area of Zanzibar with transmission levels similar to those of our second-lowest transmission setting (Kilimanjaro 600–1,200 m), little clear change in clinical incidence was seen during 2004 after the introduction of ACT, during which time 34,724 doses of artemether-lumefantrine were dispensed to a population of ∼85,000 [14]. We cannot be sure what coverage of ACT this achieved (i.e. what proportion of all antimalarials used by the population were ACT), but in our equivalent setting this would amount to ∼30% coverage and a potential ∼16% reduction in clinical episodes (Figure 3B), which could have been masked by the seasonal trends in Zanzibar or diminished by imperfect patient adherence. As well as comparing our results to studies of ACT impact, it is interesting to compare with previous introduction of long-acting nonartemisinins, which received less attention in terms of transmission reductions. For example our model suggests that when SP was first widely introduced as an efficacious drug to replace failing chloroquine treatment, there could have been a 20%–30% reduction in clinical episodes in higher-transmission areas (Table 4) due to its long prophylactic time. Documented decreases of >40% in clinical episodes or hospital admissions have coincided with SP introduction in areas of intense transmission in southern Tanzania [55] and on the coast of Kenya [45,56], although as with ACT these studies were observational and took place in the context of increasing vector control. Cluster-randomized trials measuring the transmission impact of different antimalarials used as first line treatment would be valuable in order to confirm observational evidence and the findings of our model.

With the current interest in reducing malaria transmission with a view to elimination of the parasite [57], our results suggest that ACT can be a valuable tool as part of a larger programme of control interventions, particularly in lower-transmission settings. We demonstrate that the choice of appropriate ACT partner drugs or alternative first-line treatments for a given transmission setting could play an important role in transmission control. Antimalarial properties therefore need to be taken into account in future drug development and at a national and international level in determining treatment policies if substantial reductions are to be achieved in transmission and morbidity from malaria across endemic countries.

## Supporting Information

Text S1 Extended Methods and Sensitivity Analysis (866 KB DOC)

## Abbreviations

ACT: artemisinin combination therapy
AL: artemether-lumefantrine
AQ: amodiaquine
AS: artesunate
ITN: insecticide-treated net
Pf: Plasmodium falciparum
SP: sulfadoxine-pyrimethamine

## References

[pmed-0050226-b001] Bosman A, Mendis KN (2007). A major transition in malaria treatment: the adoption and deployment of artemisinin-based combination therapies. Am J Trop Med Hyg.

[pmed-0050226-b002] Bjorkman A, Bhattarai A (2005). Public health impact of drug resistant Plasmodium falciparum malaria. Acta Trop.

[pmed-0050226-b003] Bousema JT, Schneider P, Gouagna LC, Drakeley CJ, Tostmann A (2006). Moderate effect of artemisinin-based combination therapy on transmission of Plasmodium falciparum. J Infect Dis.

[pmed-0050226-b004] Drakeley CJ, Jawara M, Targett GA, Walraven G, Obisike U (2004). Addition of artesunate to chloroquine for treatment of Plasmodium falciparum malaria in Gambian children causes a significant but short-lived reduction in infectiousness for mosquitoes. Trop Med Int Health.

[pmed-0050226-b005] Sutherland CJ, Ord R, Dunyo S, Jawara M, Drakeley CJ (2005). Reduction of malaria transmission to anopheles mosquitoes with a six-dose regimen of co-artemether. PLoS Med.

[pmed-0050226-b006] Targett G, Drakeley C, Jawara M, von Seidlein L, Coleman R (2001). Artesunate reduces but does not prevent posttreatment transmission of Plasmodium falciparum to Anopheles gambiae. J Infect Dis.

[pmed-0050226-b007] WHO (2006). RBM factsheet 9: Facts on ACTs.

[pmed-0050226-b008] Feachem R, Sabot O (2008). A new global malaria eradication strategy. Lancet.

[pmed-0050226-b009] Carrara VI, Sirilak S, Thonglairuam J, Rojanawatsirivet C, Proux S (2006). Deployment of early diagnosis and mefloquine-artesunate treatment of falciparum malaria in Thailand: The Tak Malaria Initiative. PLoS Med.

[pmed-0050226-b010] Price RN, Nosten F, Luxemburger C, ter Kuile FO, Paiphun L (1996). Effects of artemisinin derivatives on malaria transmissibility. Lancet.

[pmed-0050226-b011] Van Nam N, de Vries PJ, Van Toi L, Nagelkerke N (2005). Malaria control in Vietnam: The Binh Thuan experience. Trop Med Int Health.

[pmed-0050226-b012] Sochantha T, Hewitt S, Nguon C, Okell L, Alexander N (2006). Insecticide-treated bednets for the prevention of Plasmodium falciparum malaria in Cambodia: A cluster-randomized trial. Trop Med Int Health.

[pmed-0050226-b013] Barnes KI, Durrheim DN, Little F, Jackson A, Mehta U (2005). Effect of artemether-lumefantrine policy and improved vector control on malaria burden in KwaZulu-Natal, South Africa. PLoS Med.

[pmed-0050226-b014] Bhattarai A, Ali AS, Kachur SP, Martensson A, Abbas AK (2007). Impact of artemisinin-based combination therapy and insecticide-treated nets on malaria burden in Zanzibar. PLoS Med.

[pmed-0050226-b015] Lusingu JP, Vestergaard LS, Mmbando BP, Drakeley CJ, Jones C (2004). Malaria morbidity and immunity among residents of villages with different Plasmodium falciparum transmission intensity in North-Eastern Tanzania. Malar J.

[pmed-0050226-b016] Smith T, Beck HP, Kitua A, Mwankusye S, Felger I (1999). Age dependence of the multiplicity of Plasmodium falciparum infections and of other malariological indices in an area of high endemicity. Trans R Soc Trop Med Hyg.

[pmed-0050226-b017] Tediosi F, Maire N, Smith T, Hutton G, Utzinger J (2006). An approach to model the costs and effects of case management of Plasmodium falciparum malaria in sub-Saharan Africa. Am J Trop Med Hyg.

[pmed-0050226-b018] Yeung S, Van Damme W, Socheat D, White NJ, Mills A (2008). Cost of increasing access to artemisinin combination therapy: The Cambodian experience. Malar J.

[pmed-0050226-b019] Gelb MH (2007). Drug discovery for malaria: A very challenging and timely endeavor. Curr Opin Chem Biol.

[pmed-0050226-b020] Kumar N, Zheng H (1990). Stage-specific gametocytocidal effect in vitro of the antimalaria drug qinghaosu on Plasmodium falciparum. Parasitol Res.

[pmed-0050226-b021] Bukirwa H, Critchley J (2006). Sulfadoxine-pyrimethamine plus artesunate versus sulfadoxine-pyrimethamine plus amodiaquine for treating uncomplicated malaria. Cochrane Database Syst Rev.

[pmed-0050226-b022] Drakeley CJ, Carneiro I, Reyburn H, Malima R, Lusingu JP (2005). Altitude-dependent and -independent variations in Plasmodium falciparum prevalence in northeastern Tanzania. J Infect Dis.

[pmed-0050226-b023] Bodker R, Akida J, Shayo D, Kisinza W, Msangeni HA (2003). Relationship between altitude and intensity of malaria transmission in the Usambara Mountains, Tanzania. J Med Entomol.

[pmed-0050226-b024] Chandler CI, Drakeley CJ, Reyburn H, Carneiro I (2006). The effect of altitude on parasite density case definitions for malaria in northeastern Tanzania. Trop Med Int Health.

[pmed-0050226-b025] Pearce RJ, Drakeley C, Chandramohan D, Mosha F, Roper C (2003). Molecular determination of point mutation haplotypes in the dihydrofolate reductase and dihydropteroate synthase of Plasmodium falciparum in three districts of northern Tanzania. Antimicrob Agents Chemother.

[pmed-0050226-b026] Watkins WM, Mberu EK, Winstanley PA, Plowe CV (1997). The efficacy of antifolate antimalarial combinations in Africa: A predictive model based on pharmacodynamic and pharmacokinetic analyses. Parasitol Today.

[pmed-0050226-b027] Collins WE, Jeffery GM (2003). A retrospective examination of mosquito infection on humans infected with Plasmodium falciparum. Am J Trop Med Hyg.

[pmed-0050226-b028] Aron JL, May RM, Anderson RA (1982). The population dynamics of malaria. The population dynamics of infectious diseases: theory and applications.

[pmed-0050226-b029] Collins WE, Jeffery GM (1999). A retrospective examination of sporozoite- and trophozoite-induced infections with Plasmodium falciparum: Development of parasitologic and clinical immunity during primary infection. Am J Trop Med Hyg.

[pmed-0050226-b030] Missinou MA, Kun JF, Lell B, Kremsner PG (2001). Change in Plasmodium falciparum genotype during successive malaria episodes in Gabonese children. Parasitol Res.

[pmed-0050226-b031] Woolhouse ME, Dye C, Etard JF, Smith T, Charlwood JD (1997). Heterogeneities in the transmission of infectious agents: Implications for the design of control programs. Proc Natl Acad Sci U S A.

[pmed-0050226-b032] Smith T, Maire N, Dietz K, Killeen GF, Vounatsou P (2006). Relationship between the entomologic inoculation rate and the force of infection for Plasmodium falciparum malaria. Am J Trop Med Hyg.

[pmed-0050226-b033] Baird JK, Owusu Agyei S, Utz GC, Koram K, Barcus MJ (2002). Seasonal malaria attack rates in infants and young children in northern Ghana. Am J Trop Med Hyg.

[pmed-0050226-b034] Owusu-Agyei S, Koram KA, Baird JK, Utz GC, Binka FN (2001). Incidence of symptomatic and asymptomatic Plasmodium falciparum infection following curative therapy in adult residents of northern Ghana. Am J Trop Med Hyg.

[pmed-0050226-b035] Sama W, Owusu-Agyei S, Felger I, Dietz K, Smith T (2006). Age and seasonal variation in the transition rates and detectability of Plasmodium falciparum malaria. Parasitology.

[pmed-0050226-b036] Bonnet S, Gouagna LC, Paul RE, Safeukui I, Meunier JY (2003). Estimation of malaria transmission from humans to mosquitoes in two neighbouring villages in south Cameroon: Evaluation and comparison of several indices. Trans R Soc Trop Med Hyg.

[pmed-0050226-b037] Githeko AK, Brandling-Bennett AD, Beier M, Atieli F, Owaga M (1992). The reservoir of Plasmodium falciparum malaria in a holoendemic area of western Kenya. Trans R Soc Trop Med Hyg.

[pmed-0050226-b038] Struik SS, Riley EM (2004). Does malaria suffer from lack of memory. Immunol Rev.

[pmed-0050226-b039] Olliaro P, Pinoges L, Checchi F, Vaillant M, Guthmann JP (2008). Risk associated with asymptomatic parasitaemia occurring post-antimalarial treatment. Trop Med Int Health.

[pmed-0050226-b040] Okell L, Drakeley C, Ghani AC, Bousema JT, Sutherland C (2008). Reduction of transmission from malaria patients by artemisinin combination therapies: A pooled analysis of six randomized trials. Malar J.

[pmed-0050226-b041] Hay SI, Rogers DJ, Toomer JF, Snow RW (2000). Annual Plasmodium falciparum entomological inoculation rates (EIR) across Africa: Literature survey, Internet access and review. Trans R Soc Trop Med Hyg.

[pmed-0050226-b042] White NJ, Pongtavornpinyo W (2003). The de novo selection of drug-resistant malaria parasites. Proc Biol Sci.

[pmed-0050226-b043] Lengeler C (2004). Insecticide-treated bed nets and curtains for preventing malaria. Cochrane Database Syst Rev.

[pmed-0050226-b044] Smith DL, Dushoff J, Snow RW, Hay SI (2005). The entomological inoculation rate and Plasmodium falciparum infection in African children. Nature.

[pmed-0050226-b045] Gosling RD, Drakeley CJ, Mwita A, Chandramohan D (2008). Presumptive treatment of fever cases as malaria: help or hindrance for malaria control. Malar J.

[pmed-0050226-b046] Yeung S, White NJ (2005). How do patients use antimalarial drugs? A review of the evidence. Trop Med Int Health.

[pmed-0050226-b047] WHO (2006). WHO guidelines for treatment of malaria.

[pmed-0050226-b048] White NJ (2008). How antimalarial drug resistance affects post-treatment prophylaxis. Malar J.

[pmed-0050226-b049] Hastings IM, Watkins WM, White NJ (2002). The evolution of drug-resistant malaria: The role of drug elimination half-life. Philos Trans R Soc Lond B Biol Sci.

[pmed-0050226-b050] Chen PQ, Li GQ, Guo XB, He KR, Fu YX (1994). The infectivity of gametocytes of Plasmodium falciparum from patients treated with artemisinin. Chinese Med J.

[pmed-0050226-b051] El-Sayed B, El-Zaki SE, Babiker H, Gadalla N, Ageep T (2007). A randomized open-label trial of artesunate-sulfadoxine-pyrimethamine with or without primaquine for elimination of sub-microscopic P. falciparum parasitaemia and gametocyte carriage in eastern Sudan. PLoS ONE.

[pmed-0050226-b052] Gardella F, Assi S, Simon F, Bogreau H, Eggelte T (2008). Antimalarial drug use in general populations of tropical Africa. Malar J.

[pmed-0050226-b053] McCombie SC (2002). Self-treatment for malaria: the evidence and methodological issues. Health Policy Plan.

[pmed-0050226-b054] National Bureau of Statistics of the United Republic of Tanzania (2003). Tanzania 2002 population and housing census.

[pmed-0050226-b055] Schellenberg D, Menendez C, Aponte J, Guinovart C, Mshinda H (2004). The changing epidemiology of malaria in Ifakara Town, southern Tanzania. Trop Med Int Health.

[pmed-0050226-b056] Okiro EA, Hay SI, Gikandi PW, Sharif SK, Noor AM (2007). The decline in paediatric malaria admissions on the coast of Kenya. Malar J.

[pmed-0050226-b057] [No authors listed] (2007). Is malaria eradication possible. Lancet.

[pmed-0050226-b058] Falk N, Maire N, Sama W, Owusu-Agyei S, Smith T (2006). Comparison of PCR-rflp and genescan-based genotyping for analyzing infection dynamics of Plasmodium falciparum. Am J Trop Med Hyg.

[pmed-0050226-b059] Sama W, Dietz K, Smith T (2006). Distribution of survival times of deliberate Plasmodium falciparum infections in tertiary syphilis patients. Trans R Soc Trop Med Hyg.

[pmed-0050226-b060] Eyles DE, Young MD (1951). The duration of untreated or inadequately treated Plasmodium falciparum infections in the human host. J Natl Malar Soc.

[pmed-0050226-b061] Smith T, Charlwood JD, Takken W, Tanner M, Spiegelhalter DJ (1995). Mapping the densities of malaria vectors within a single village. Acta Trop.

[pmed-0050226-b062] Miller MJ (1958). Observations on the natural history of malaria in the semi-resistant West African. Trans R Soc Trop Med Hyg.

[pmed-0050226-b063] Bruce-Chwatt LJ (1963). A longitudinal survey of natural malaria infection in a group of West African adults. I. West Afr Med J.

[pmed-0050226-b064] Jeffery GM, Eyles DE (1954). The duration in the human host of infections with a Panama strain of Plasmodium falciparum. Am J Trop Med Hyg.

[pmed-0050226-b065] Ruebush TK, Kern MK, Campbell CC, Oloo AJ (1995). Self-treatment of malaria in a rural area of western Kenya. Bull World Health Organ.

[pmed-0050226-b066] Espino F, Manderson L (2000). Treatment seeking for malaria in Morong, Bataan, the Philippines. Soc Sci Med.

[pmed-0050226-b067] Tarning J, Ashley EA, Lindegardh N, Stepniewska K, Phaiphun L (2008). Population pharmacokinetics of piperaquine after two different treatment regimens with dihydroartemisinin-piperaquine in patients with Plasmodium falciparum malaria in Thailand. Antimicrob Agents Chemother.

[pmed-0050226-b068] Ezzet F, van Vugt M, Nosten F, Looareesuwan S, White NJ (2000). Pharmacokinetics and pharmacodynamics of lumefantrine (benflumetol) in acute falciparum malaria. Antimicrob Agents Chemother.

