# Supplementary material for: Modelling the Impact of Artemisinin Combination Therapy and Long-Acting Treatments on Malaria Transmission Intensity
Source: PLoS Med. 2008 Nov 25;5(11):e226. doi: 10.1371/journal.pmed.0050226 (PMC2586356; doi:10.1371/journal.pmed.0050226)
Supplement: Text S1 — (866 KB DOC) [file pmed.0050226.sd001.doc]

Text S1. Extended Methods & Sensitivity Analysis

**Extended methods**

*Basic human transmission model in the absence of treatment*

We model parasite transmission in the human and mosquito populations using a deterministic compartmental structure. All parameters are shown in Table I. In the absence of treatment, at each time *t* humans in age-group *i* can be in one of three states: susceptible *S(i,t)*, latent *E(i,t)*, or infectious *I(i,t)*. Susceptible individuals in age group *i* become infected at rate Λ*(i,t)*=*mab(i)I_M_(t)* where *m* is the density of mosquitoes, *a* is the human biting rate, *I_M_* is the prevalence of infection in the mosquito population and *b(i)* is the probability of developing blood stage infection in age group *i*. The latent period is divided into two stages denoted *E_j_(i,t)*; during the first the parasites are liver- and early blood-stage and are subpatent (i.e. not detectable on a blood slide), and during the second stage the infection becomes patent. The infectious period is divided into 4 stages (denoted *I_j_(i,t)*) with infectiousness greatest in the first two stages and lower in subsequent stages to replicate patterns observed in longitudinal human-to-mosquito transmission experiments (Table I ) [1]. We assume that an untreated infection is patent during the first 3 stages then becomes subpatent, similarly to previous models [2]. From the fourth stage of the infectious period, individuals recover to the susceptible state. Superinfections occur in infectious individuals independently of the initial infection at a rate *Λ(i,t)* and upon superinfection individuals return to the first stage of the infectious period, extending the overall duration of parasitaemia. This superinfection mechanism differs from that used in previous models [3] but gives almost identical quantitative results.

The equations describing transmission among the human population in the absence of treatment, ignoring explicit representation of ageing, are given by the following set of differential equations:

$$\begin{matrix} \frac{dS(i)}{dt}=r_{4}(i)I_{4}(i)-\Lambda(i,t)S(i) \\ \frac{dE_{1}(i)}{dt}=\Lambda(i,t)S(i)-\tau_{1}E_{1}(i) \\ \frac{dE_{2}(i)}{dt}=\tau_{1}E_{1}(i)-\tau_{2}E_{2}(i) \\ \frac{dI_{1}(i)}{dt}=\tau_{2}E_{2}(i)+\sum_{j=1}^{4} \Lambda(i,t)I_{j}(i)-\Lambda(i,t)I_{1}(i)-r_{1}(i)I_{1}(i) \\ \frac{dI_{j}(i)}{dt}=r_{j-1}I_{j-1}(i)-r_{j}(i)I_{j}(i)-\Lambda(i,t)I_{j}(i)j=2,3,4 \end{matrix}$$

where $1/\tau_{j}$ is the duration of latent stage *j* and $1/r_{j}(i)$ is the duration of infectious stage *j* in age group *i*. This is shown schematically in Figure I.

**Figure I. Model schematic showing transmission in humans in a single age group in the absence of treatment.**

I_2_

S

E_1_

I_1_

I_3_

I_4_

E_2_

τ_2_

Λ

Λ

Λ

Λ

Λ

τ_1_

r_4_

r_1_

r_2_

r_3_

*Treatment of Symptomatic Infection*

We assume that a proportion of new infections will be symptomatic and that symptoms occur at the beginning of an infection followed by a longer period of asymptomatic parasitaemia. This is supported by data from experimental infections [4], seasonal patterns in which parasitaemia but not clinical attacks persists through seasons of absent transmission [5] and molecular studies in which symptoms coincide with the appearance of a new parasite clone [6,7]. Existing parasitaemia does not prevent symptoms in a superinfection, although it may make them less likely via immune or competitive mechanisms [8,9]. For simplicity we assume that the proportion of superinfections resulting in symptoms is the same as for infections occurring in susceptibles.

A constant proportion *f(i)* of symptomatic individuals are treated (although this may vary by age – see main text, methods, model parameterization and validation). Treatment can reduce transmission in three main ways in the model: by reducing the duration for which treated individuals are infectious (length of time gametocytes remain in the bloodstream), reducing their infectiousness (density and infectivity of gametocytes) and providing prophylaxis. The efficacy of an antimalarial also determines its impact on transmission. In studies of treated individuals, asexual densities appear to be reduced swiftly according to blood slides, however gametocytaemia and infectiousness persist for longer [10]. In our model, the infectious period of treated individuals *T* is divided into four stages as for untreated individuals. Rates of progression through these stages are faster (Table I) and the infection is assumed to become subpatent after the first stage. In the case of gametocytocidal antimalarials, infectiousness is reduced by a constant proportion throughout the infectious period relative to untreated individuals. The total duration of effective prophylaxis is equal to the duration of minimum inhibitory antimalarial concentrations in the blood minus the duration of liver stage infection, since current, widely-used antimalarials only protect against blood stage parasites. We assume 100% protection from infection during this time. This includes protection from superinfection during the infectious period, and if prophylactic effects persist after parasites are cleared (depending on the antimalarial being used and age-specific recovery rate), individuals enter the protected state *P(i,t)* before returning to the susceptible state *S(i,t)*. Treatment is efficacious at clearing parasitaemia with a probability *h*, which is equal to the prevalence of infections consisting of parasites susceptible to the drug. The remaining treated patients suffer parasitological failure, which is incorporated by assuming no effect of treatment for simplicity, so that these patients enter the first stage of the untreated state *I_1_(i,t)*. Clinical failure, i.e. parasitological failure accompanied by symptoms, is not explicitly incorporated in the model. In our data from the Tanzania survey, individuals were asked if they had used antimalarials within the previous 14 d. Clinical failure usually occurs on average around 5 d after treatment [11], so we assume that re-treatment would occur relatively quickly after the first treatment episode. Therefore it would have little effect on the period prevalence measure of antimalarial use within the last 14 d, or the stage at which infection is treated. However, prevalence of symptomatic malaria was measured on the day of the survey only so we use a relatively high estimate of duration of symptoms (Table I) to allow for clinical failure.

The transmission cycle for symptomatic humans treated with a single type of antimalarial is shown schematically in Figure II and described by the following differential equations:

$$\begin{matrix} \frac{dS(i)}{dt}=r_{4}(i)I_{4}(i)+\left[1-\alpha(i)\right]q_{4}(i)T_{4}(i)+\psi(i)P(i)-\Lambda(i,t)S(i) \\ \frac{dE_{1}(i)}{dt}=\Lambda(i,t)S(i)-\tau_{1}E_{1}(i) \\ \frac{dE_{2}(i)}{dt}=\tau_{1}E_{1}(i)-\tau_{2}E_{2}(i) \\ \frac{dI_{1}(i)}{dt}=[1-\varphi(i)f(i)h)]\left[\tau_{2}E_{2}(i)+\sum_{j=1}^{4} \Lambda(i,t)I_{j}(i)+\sum_{j=1}^{4} \rho_{j}(i)\Lambda(i,t)T_{j}(i)\right]-\Lambda(i,t)I_{1}(i)-r_{1}(i)I(i) \\ \frac{dI_{j}(i)}{dt}=r_{j-1}(i)I_{j-1}(i)-r_{j}(i)I_{j}(i)-\Lambda(i,t)I_{j}(i)j=2,3,4 \\ \frac{dT_{1}(i)}{dt}=\varphi(i)f(i)h\left[\tau_{2}E_{2}(i)+\sum_{j=1}^{4} \Lambda(i,t)I_{j}(i)+\sum_{j=1}^{4} \rho_{j}(i)\Lambda(i,t)T_{j}(i)\right]-\rho_{1}(i)\Lambda(i,t)T_{1}(i)-q_{1}(i)T_{1}(i) \\ \frac{dT_{j}(i)}{dt}=q_{j-1}(i)T_{j-1}(i)-q_{j}(i)T_{j}(i)-\rho_{j}(i)\Lambda(i,t)T_{j}(i)j=2,3,4 \\ \frac{dP(i)}{dt}=\alpha(i)q_{4}(i)T_{4}(i)-\psi(i)P(i) \end{matrix}$$

where $q_{j}(i)\geq r_{j}(i)$is the rate of transition through the infectious stages in treated infections, $0<\rho_{j}(i)<1$ is the proportion susceptible to superinfection in those receiving antimalarials (indicating whether prophylactic blood levels of antimalarial are present) and $\alpha(i)$is an indicator variable for whether there is an additional prophylactic effect of the antimalarial that extends beyond the duration of treated infection. Individuals on treatment in stage *j* of the infectious period in age group *i* are fully protected from superinfection ($\rho_{j}(i)=0$) if the mean duration of effective prophylactic time is greater than the cumulative duration of the infectious period up to the end of stage *j* in age group *i*. If the effective prophylactic time does not extend into stage *j* of the infectious period, individuals in this state are fully susceptible to superinfection, and if it partially overlaps then a proportion are susceptible. Expressed mathematically:

$$\begin{matrix} \rho_{j}(i)=\begin{matrix} 1 & if(1/\omega-1/\gamma)\leq\sum_{m=1}^{j-1} 1/q_{m}(i) & \\ 0 & if(1/\omega-1/\gamma)>\sum_{j=1}^{j} 1/q_{j}(i) & \\ 1-\text{ }\left[\text{(}1/\omega-1/\gamma-\sum_{m=1}^{j-1} 1/q_{m}(i))/(1/q_{j}(i))\right] & otherwise & \end{matrix} \\ \end{matrix}$$

where $1/\omega$is the mean duration of antimalarial blood concentrations above the minimum inhibitory concentration and 1/$\gamma$ is the mean duration of liver stage infection.

The parameter $\alpha$ is then similarly given by:

$$\alpha(i)=\begin{matrix} 0 & if(1/\omega-1/\gamma)\leq\sum_{j=1}^{4} 1/q_{j}(i) \\ 1 & otherwise \end{matrix}$$

and$\psi(i)$, the rate of losing prophylactic antimalarial levels once in a protected uninfected state *P(i,t)*, is determined by the extent to which the duration of effective prophylaxis extends beyond the total duration of the infectious period:

$$1/\psi(i)=1/\omega-1/\gamma-\sum_{j=1}^{4} 1/q_{j}(i)$$

If more than one antimalarial is in use in the modeled population, the infectious state *T(i,t)* and prophylactic state *P(i,t)* are stratified by treatment type and the parameters $q_{j}(i)$, $\rho_{j}(i)$, $\alpha(i)$ and$\psi(i)$ allowed to vary by antimalarial (see Table I). The proportion of treated individuals using ACT, *s*, rather than failing non-artemisinins varies between 0 and 1. The flow diagram (Figure II) shows the transmission cycle for only one type of antimalarial for simplicity.

**Figure II Model section showing the transmission cycle in symptomatic effectively-treated humans, for a single age and exposure group and single type of antimalarial.**

S

E_1_

E_2_

P

T_2_

T_1_

T_3_

Λ

ρ_1_Λ

τ_1_

q_1_

q_2_

q_3_

αq_4_

ψ

infections & superinfections with no or failed treatment as in Figure I

T_4_

(1-α)q_4_

ρ_2_Λ

ρ_3_Λ

ρ_4_Λ

φ*fh*

τ_2_

1-(φ*fh)*

*Presumptive treatment of asymptomatic individuals*

Asymptomatic individuals may also receive antimalarials presumptively which can alter the course of any infection present and, depending on the drug pharmacodynamics, may provide a period of prophylaxis. We assume that this treatment occurs at a constant rate $z(i)$ from all states other than those currently receiving treatment. The full model for a single antimalarial showing presumptive treatment in addition to the untreated infections and symptomatic treated infections already described is shown in the flow diagram in Figure III. In order to model prophylactic effects, the treated state *T_j_(i,t)* is divided into *k* strata *T_jk_(i,t)* according to the stage of infection at which individuals received treatment. Those newly infected (*E_1_(i,t)*, *E_2_(i,t)* and *I_1_(i,t)*) who receive presumptive treatment enter the first stage of the treated compartment *T_11_(i,t)* as do new symptomatic treated infections. Those who are already at stage 2 of the infectious period (*I_2_(i,t)* and *T_21_(i,t)*) when they receive presumptive treatment begin the period of prophylaxis from this stage. This is modeled by their entering the second strata of the treated state *T_22_(i.t)*. As their infection is already more advanced the infectiousness and remaining duration of this state is identical to those in *T_21_(i,t)*; only prophylactic effects are different. Similarly those who receive presumptive period during stage 3 of the infectious period (*I_3_(i,t), T_31_(i,t), T_32_(i,t)*) move to the state *T_33_(i,t)* and begin prophylaxis from this stage. Those receiving presumptive treatment in the final stage of the infectious period become aparasitaemic and therefore either return to the susceptible state or enter the protected state dependent on the drug being used. In a similar way, the protected, uninfected state *P(i,t)* is stratified into *j* states denoted 1-4 to allow for the different amounts of prophylactic time remaining according to the stage at which treatment was received. *P_1_(i,t), P_2_(i,t) and P_3_(i,t)* represent those treated at infectious stage 1, 2 and 3, respectively. *P_4_(i,t)* describes individuals receiving presumptive treatment while uninfected or in the final stage of the infectious period. An additional indicator variable $\sigma$ describes whether the duration of effective prophylaxis is longer than zero:

$$\sigma=\begin{matrix} 0 & if(1/\omega-1/\gamma)\leq0 \\ 1 & otherwise \end{matrix}$$

The parameters $\rho_{jk}(i)$,$\alpha_{k}(i)$and$\psi_{k}(i)$ take the same meaning as described above but are now calculated separately for each stratum *k* of treated individuals *T_jk_(i,t)* taking into account the state in which treatment was received (calculations are as above except for *k*=2, $1/q_{m}$ is summed from stage 2, for *k*=3, from stage 3 etc). Superinfection is included in the model as before but not explicitly shown on the diagram. We assume no effect of presumptive treatment in cases of parasitological failure so that individuals do not change state.

The differential equations for the full model incorporating treatment of symptomatic infections and presumptive treatment of asymptomatic infections with a single antimalarial are given below and the model is shown schematically in Figure III.

**Figure III. Full model showing transmission cycle with presumptive treatment (green, dashed lines), infections that are untreated, that fail treatment or that are symptomatic and treated, as before (Figures I-II) for a single antimalarial and age group. The treated, infectious state *T(i,t)* and protected (by prophylaxis), uninfected state *P(i,t)* are stratified according to the stage at which treatment was received. Superinfections are incorporated as in Figures I-II but not shown in detail here for simplicity (see equations).**

*Age and Heterogeneity in Exposure*

The model is stratified into three age groups (0-4 yrs, 5-14 yrs and 15+ yrs) to match the data with a birth rate equal to the total death rate resulting in a constant population size. We assume a constant death rate from the first two age categories and a higher death rate from the last, which results in a constant age distribution closely matching that of the Tanzanian 2002 census [12]. Malaria-attributable deaths are not explicitly incorporated. We allow for heterogeneity of exposure by additionally stratifying each age group into two exposure levels such that a proportion of the population $\kappa$are highly exposed and are subject to a force of infection $\Lambda(i,t)=m\theta ab(i)I_{M}(t)$, where$\theta$ is the proportion of mosquito bites received by the high exposure group and the other parameters are as defined earlier. The force of infection experienced by the proportion of the population 1-$\kappa$in the low exposure group is $\Lambda(i,t)=m(1-\theta)ab(i)I_{M}(t)$[13,14]. Immunity is modelled by incorporating age-dependency in the probability that an infectious bite develops into a blood stage infection, *b(i)* [15], the proportion of infections which become symptomatic *(i)* [16,17], the rate that an infection progresses to the final subpatent stage *r_3_(i)* (but not the overall duration of infection [18]), and infectiousness to mosquitoes *c_j_ (i)* [19,20]. The parameters *b(i)* and *(i)* are allowed to vary with altitude, but average *c_j_(i)* over the full course of an infection and across age groups, and average *r_3_(i)* across age groups are kept constant with the parameters in older age-groups (5-14 years and 15+ years) scaled from the baseline value in the youngest age-group (0-4 years) (see Table I). During model runs, average infectiousness of infected individuals in a population increases with transmission intensity because the higher rate of superinfections increases the chance they are in the early stage of infection. We assume negligible short-term change in immunity so that these parameters do not vary over time [21]. In addition we allow for an increase in exposure to biting with age due to larger body surface area, such that each age group is subject to *v*(i) times the rate of biting relative to the youngest age group (as previously modeled [15]).The total contribution of each age-group to the infectious reservoir is then given by

$$L(i,t)=w(i)v(i)\sum_{j=1}^{4} \sum_{k=j}^{3} (I_{j}(i,t)+eT_{jk}(i,t))c_{j}(i)$$

where *w(i)* is the proportion of the population in age-group *i* and *c_j_(i)* is the infectivity of an infectious individual of age *i* in stage *j* of the infectious period and *e* is the reduction in infectiousness caused by gametocytocidal treatment if present.

*Vector Dynamics*

Transmission among mosquitoes is incorporated dynamically and responds to changes in the prevalence of infectious humans. Mosquitoes are born into a susceptible state *S_M_* and if they become infected they enter a latent state *E_M_* before becoming infectious *I_M_*. A constant death rate and population size is assumed. There is no clearance of infection before death. The transitions between these states are described by:

$$\begin{matrix} \frac{dS_{M}}{dt}=g-\sum_{i} L(i)aS_{M}-gS_{M} \\ \frac{dE_{M}}{dt}=\sum_{i} L(i)aS_{M}-\tau_{M}E_{M}-gE_{M} \\ \frac{dI_{M}}{dt}=\tau_{M}E_{M}-gI_{M} \end{matrix}$$

where *g* is the birth and death rate and$\tau_{M}$ is the rate at which mosquitoes become infectious once infected.

*Model parameterization and validation*

All details are as given in the main text. The model was fitted to the observed prevalence of slide-positive infection by age group for each setting (Table II). In the data, the oldest age group sampled was 15-45 years but our model includes all individuals over 45 years. We assume that those over 45 years have the same characteristics as the 15-45 year olds.

**Table I. Parameters*.***

| *Parameters* | *Definitions & units* | *Estimates (data/literature)* | *Values used in model* |
| --- | --- | --- | --- |
| **Transmission cycle parameters** | | | |
| *m* | Ratio female mosquitoes: humans. | 0-500 [22] | varied to fit data |
| *a* | Human bloodmeals taken per mosquito per d | 0.02 – 0.465 [23,24] | 0.125 |
| *b* | Probability of human blood stage infection per infected mosquito bite | 0.3-0.5 [25] | 0.2 |
|  |  | 0.03-0.13 [26] | or varied to fit data |
| *1/τ_M_* | Duration of mosquito latent period, d (from ingestion of gametocytes to appearance of sporozoites in salivary glands) | 9-10 at 28ºC [27] | 10 |
| *1/g* | Mosquito lifespan, d | 1/0.06 [28] | 1/0.1 |
|  |  | 1/0.16 [29] |  |
| *1/τ_1_* | Time from sporozoite inoculation to development of patent blood infection, d | 7-13 [30] | 10 |
| *1/τ_2_* | Time from patent blood infection to infectious stage, d | 10 [31] | 10 |
| *1/γ* | Time from sporozoite inoculation to development of blood stage infection, d | 7 | 7 |
| κ | Proportion of the human population in high exposure stratum who receive a proportion of mosquito bites θ | 0.10-0.15 [13,14] | 0.10 |
| θ | % mosquito bites received by the high exposure stratum of the human population | 0.8 [13,14] | 0.8 |
| $\sum_{j=1}^{4} 1/r_{j}$ | Mean duration of infectious period in untreated humans, d |  |  |
|  | total | 210 [32] | 210 |
|  |  | 210 [33] |  |
| *1/r_1_,… 1/r_j_* | proportion of time spent in stage 1.. *j* of untreated infectious period | 10, 10, 60, 120 in non-immunes [1] | 10.5, 10.5, 31.5, 157.5 |
| **Parameters which vary by age** | | | |
| $\sum_{j}^{3} 1/r_{j}$ | Duration of patent infection in untreated humans, d |  |  |
|  | average | 121 non immunes [30] | 50 |
|  | in age groups 2,3 as a fraction of the duration in the youngest age group 1 | ~0.1 in age group 44+ yrs relative to 1-4yrs [34] | 0.33, 0.2 |
|  |  |  |  |

| *Parameters* | *Definitions & units* | *Estimates (data/literature)* | *Values used in model* |
| --- | --- | --- | --- |
| *c’* | Probability of transmission from an untreated infectious human to a mosquito, per bite |  |  |
|  |  |  |  |
|  | Population average | 0.115 [35] | See *c* |
|  |  | 0.078 [20] |  |
| *c* | average | See *c’* | 0.05 |
| *p(i)* | relative infectiousness in age groups 1..i | Calculated from gametocyte density in Table II | *see Table II* |
| *q_1_...q_j_* | relative infectiousness in infectious stage 1…j | Approximated from [1] | 1.90, 3.08, 1.53, 0.28 |
| *c_j_(i)* | *c_j_(i)*= *p(i)q_j_c* |  |  |
| *v(i)* | Relative rate of mosquito biting in each age group due to body surface area. |  |  |
|  | Age group 1, 2, 3 | 0.52, 0.96, 1.52 | 0.52, 0.96, 1.52 |
|  |  | Calculated from [15] |  |
| **Treatment parameters** | | | |
| φ | Probability of developing symptoms upon infection | 0.20 [16] | Calculated from data in Table II & model |
|  |  | 0.31-0.34 [17] |  |
| Ω | Duration of symptomatic episode of malaria, d | 4.2 [36] | 10 |
|  |  | 2.6 [37] |  |
|  |  | 10.7-13.1 [4] |  |
| *f* | Proportion of symptomatic cases treated with an antimalarial | 0.46-0.60 for ‘febrile illness’ [38] | maximum 0.8 |
|  |  | 0.78 [39] |  |
| *s* | Proportion of treatments which are ACT | - | 0-1 |
| $\sum_{j=1}^{4} 1/q_{j}$ | Duration of gametocytaemia in treated infection, d |  |  |
|  | non-artemisinin | SP-AQ 66.4 [unpublished analysis] | 66 |
|  | ACT | SP-AS 14.9 [unpublished analysis] | 15 |
|  |  | AL 6.3  [unpublished analysis] |  |
| *h* | Proportion with no parasitological treatment failure in those treated with non-artemisinins | <40% prevalence of parasites without double-triple SP-resistant genotype in study area [40] | 50 |

| *Parameters* | *Definitions & units* | *Estimates (data/literature)* | *Values used in model* |
| --- | --- | --- | --- |
| *e* | Relative infectiousness of individuals treated with ACT compared to non-artemisinins / untreated | 0.68 [10] | 0.68 |
| 1/$\omega$ | Duration of inhibitory antimalarial blood concentration, d |  |  |
|  | non-artemisinin (efficacious) | 20 piperaquine [41]  >52 SP, fully sensitive strain [42] | 25 |
|  | non-artemisinin (pre-ACT with treatment failure) | 7 SP N51I, S108N mutant, partially resistant [42] | 7 |
|  | ACT (partner drug) | 8.5-12.4 lumefantrine [43] | 10 |
| *z(i)* | Rate of presumptive treatment in the general population in age group i (patent vs no infection) | Calculated from data in Table II |  |
| **Demographic parameters** | | | |
| *1/μ’* | Human life expectancy, years | 47.1 [44] | 47.1 |
| *μ(i)* | Death rate per year in age group *i*  age groups 1 and 2  age group 3 | *see 1/ μ’*  set to approximate *w(i)* values in [12] | 0.021  0.054 |
| *w(i)* | Proportion of population in age groups 1,2,3 | 0.165, 0.278, 0.558 | 0.174, 0.288 , 0.538 |
|  |  |  | Calculated from *μ(i)* and *ν(i)* |
| *1/ν(i)* | Time spent in age groups 1,2, years | - | 5,10 |

**Table II. Survey data from six transmission settings in Tanzania in order of decreasing prevalence of infection. To characterize pre-ACT scenarios, the model was fitted to the prevalence of slide-positive infection, symptomatic malaria and history of antimalarial use by age and area, and gametocyte density data were used to parameterize the relative infectiousness of each age group in each area.**

| Region | Altitude band (m) & age group | N | *Pf* slide-positive (%) | Prevalence symptomatic infections* (% of population) | Gametocyte density/μl ^‡^ (arithmetic mean) | Self-report use of antimalarials in past 14 d (%) |
| --- | --- | --- | --- | --- | --- | --- |
|  |  |  |  |  |  |  |
| Tanga | <600m |  |  |  |  |  |
|  | **all** | **748** | **57.1** | **8.13** | **17.4** | **10.6** |
|  | <5 yrs | 216 | 66.7 | 10.95 | 41.9 | 19.1 |
|  | 5-14 yrs | 250 | 70.4 | 10.53 | 5.8 | 7.6 |
|  | 15-45 yrs | 282 | 37.9 | 3.91 | 3.4 | 6.8 |
| Tanga | 600-1200m |  |  |  |  |  |
|  | **all** | **961** | **30.1** | **6.62** | **17.1** | **6.5** |
|  | <5 yrs | 258 | 28.7 | 7.09 | 40.5 | 7.5 |
|  | 5-14 yrs | 343 | 37.3 | 9.41 | 11.5 | 5.9 |
|  | 15-45 yrs | 360 | 24.2 | 3.63 | 5.4 | 6.2 |
| Tanga | >1200m |  |  |  |  |  |
|  | **all** | **1149** | **21.4** | **2.9** | **24.1** | **2.9** |
|  | <5 yrs | 326 | 19.3 | 3.72 | 55.2 | 3.4 |
|  | 5-14 yrs | 415 | 26.3 | 3.41 | 15.3 | 2.5 |
|  | 15-45 yrs | 408 | 18.1 | 1.73 | 10.5 | 3.0 |
| Kilimanjaro | <600m |  |  |  |  |  |
|  | **all** | **249** | **18.1** | **3.61** | **6.8** | **6.1** |
|  | <5 yrs | 67 | 7.5 | 1.49 | 5.3 | 7.6 |
|  | 5-14 yrs | 92 | 33.7 | 5.43 | 8.6 | 8.7 |
|  | 15-45 yrs | 90 | 10.0 | 3.33 | 1.5**†** | 2.2 |
| Kilimanjaro | 600-1200m |  |  |  |  |  |
|  | **all** | **1441** | **10.7** | **0.35** | **9.4** | **5.5** |
|  | <5 yrs | 423 | 9.5 | 0.24 | 34.0 | 8.5 |
|  | 5-14 yrs | 485 | 12.8 | 0.62 | 1.3 | 2.9 |
|  | 15-45 yrs | 533 | 9.8 | 0.19 | 0.3**†** | 5.5 |
| Kilimanjaro | >1200m |  |  |  |  |  |
|  | **all** | **1119** | **3.7** | **0.27** | **4.6** | **2.4** |
|  | <5 yrs | 295 | 2.4 | 0.34**†** | 1.9**†** | 2.8 |
|  | 5-14 yrs | 402 | 5.0 | 0.25 | 0.7**†** | 1.0 |
|  | 15-45 yrs | 422 | 3.3 | 0.24**†** | 11.4 | 3.3 |

* fever>37.5°C + *Pf* density> age- and altitude-specific threshold calculated in [45]

**†** True value = 0, given value = 0.5 x detection threshold (where detection thresholds are 1 symptomatic case, and 1 individual with 26.7 gametocytes/μl among slide-positives).

^‡^ Among those who are positive for any parasite stages

**Sensitivity analysis**

In order to further explore underlying relationships between treatment and transmission reduction and to test the robustness of the model predictions to our assumptions we repeated our main simulations varying key parameters and parts of the model structure.

First we show results from a simplified version of our model. This did not use the survey data and ignored presumptive treatment, treatment failure, age and immune effects, and variations in infectiousness over the course of infection, but in other respects was the same as the original model. We assumed the same, constant percentage of 50% of incident infections were treated in all simulations, and compared the impact of antimalarials to a scenario without any treatment. Figure IV shows the relationship between pre-treatment slide-prevalence and the percentage reduction in infection incidence achieved by antimalarials with gametocytocidal or prophylactic effects. While the predicted size of the impact differs to some extent from the results of the main model, the same trends are clear: impact of gametocytocidal antimalarials decreases with baseline slide-prevalence and the impact of prophylactic antimalarials increases. These results show both that these trends are robust to assumptions, and also help to elucidate reasons for the variation in impact of gametocytocidal drugs across transmission settings. In previous results the relative impact of ACT correlated well with the ratio of treatment rate to the infection rate (Figure 3a main text), which decreased as pre-ACT transmission became higher. In these simulations however, we kept this ratio constant at 0.5 but still see a similar variation, indicating the treatment to infection ratio cannot be the only driving factor. This highlights further underlying differences in transmission dynamics between high and low prevalence settings. Human and mosquito prevalence at equilibrium are determined essentially by the ratio of the infection rate relative to the rate of losing infection (*x*) through the relationship *I ≈* Λ / (Λ+*x*) where Λ is the incidence of infection in humans or mosquitoes. If Λ>>*x* then increasing the recovery speed *x* in humans or reducing the incidence of infection Λ on mosquitoes (via reduction in human infectiousness) will have little impact whereas when the infection rate$\Lambda$is more equal to the rate of losing infection, reductions in Λ or *x* will reduce the prevalence in both mosquitoes and humans more rapidly.

**Figure IV. Impact of varying antimalarial pharmacodynamic properties on infection incidence in a simplified version of the model. Data, age, immunity & varying infectiousness over time are not incorporated, and 50% of infections are treated in all settings. Treatment with 100% efficacy and (a) gametocytocidal properties only (% reduction in both duration of infection and average infectiousness over time are shown) and (b) prophylactic effects only are compared to a scenario without antimalarial treatment.**

In addition to using a simplified version of the model, we also explored the effects of varying key parameters in the main model. We reproduce the main results of the model using a maximum and minimum value for each parameter (Table III) while keeping other parameters fixed, showing impact of ACT and an efficacious non-gametocytocidal antimalarial with a long prophylactic time. The model was re-fitted to the baseline data in the six survey areas for each new parameter combination, then the ACT or long-acting drug were introduced. Figures V-VII show the results of these sensitivity analyses. The parameters alter the results in one or both of the following ways: 1) by altering incidence rates of infection and symptomatic episodes estimated from prevalence data in the pre-ACT scenario and 2) by affecting the ability of ACT/long-acting treatment to reduce transmission. In several of these analyses the estimated rate of clinical episodes in the pre-ACT scenario was higher than the estimated incidence of infection, which was assumed to represent an unrealistic combination of parameters. The occurrence of these results is reasonable given that in some low transmission settings or young age groups the percentage of infections causing symptoms may approach 100%, so that even small errors in parameter estimates could increase it beyond this limit. These data points are not shown, and those results in other settings using the same parameter combination are shown as points only without a connecting line to indicate uncertainty.

**Table III. Sensitivity analysis: key variables, parameters varied to change these, best estimates used to produce results in the main paper,** low and high estimates.

| Variables | Parameters | Estimate types | Values |
| --- | --- | --- | --- |
| Proportion of symptomatic cases treated | *f* | low  best  high | 0.5  0.8  1.0 |
| Duration of symptomatic episode of malaria, d | $\Omega$ | low  best  high | 5  10  15 |
| Mean duration of infectious period in untreated humans, d | $1/\sum_{j}^{3} r_{j}$ Duration of patent infection $1/\sum_{j}^{4} r_{j}$ Total duration of infectiousness. | low  best  high | $1/\sum_{j=1}^{3} r_{j}$=30, $1/\sum_{j=1}^{4} r_{j}$=150  $1/\sum_{j=1}^{3} r_{j}$=50, $1/\sum_{j=1}^{4} r_{j}$=210  $1/\sum_{j=1}^{3} r_{j}$=70, $1/\sum_{j=1}^{4} r_{j}$=250 |
| Heterogeneity of exposure | κ Proportion of the human population in high exposure stratum who receive a proportion of mosquito bites θ | low  best  high | κ=20, θ=70  κ=10, θ=80  κ=5, θ=90 |
| Impact size of pre-ACT non-artemisinin | *h* % treatments efficacious $1/\sum_{j=1}^{4} q_{j}$ Duration of gametocytaemia in treated infection, d  1/$\omega$ Duration of inhibitory antimalarial blood concentration, d | low  best  high | *h*=0.25, $1/\sum_{j=1}^{4} q_{j}$=147, 1/$\omega$= 5  *h*=0.50, $1/\sum_{j=1}^{4} q_{j}$=66, 1/$\omega$= 7  *h*=0.65, $1/\sum_{j=1}^{4} q_{j}$=32, 1/$\omega$= 15 |
| Age group variation in infectiousness, recovery rates, infection rates, proportion of infections becoming symptomatic | Number of age groups | No variation  With variation | 1 (population average values used)  3 (age-group-specific values used) |
| Impact size of ACT | *e* Relative infectiousness of individuals treated with ACT  1/$\omega$ Duration of inhibitory antimalarial blood concentration, d,  $\sum_{j=1}^{4} 1/q_{j}$Duration of treated infection, d | Low  Best  High | *e* =1, 1/$\omega$=5, $\sum_{j=1}^{4} 1/q_{j}$=30  *e* =0.68, 1/$\omega$=10, $\sum_{j=1}^{4} 1/q_{j}$=15  *e* =0.5, 1/$\omega$=25, $\sum_{j=1}^{4} 1/q_{j}$=5 |

Of the parameters explored, changing the degree of exposure heterogeneity in the population has one of the largest impacts on the results (Figure V). Having infection concentrated in a smaller group of more highly exposed people (5% of people receiving 90% of mosquito bites) increases pre-ACT incidence of infection estimates, so that it is harder to impact on transmission (see figure IV above). In a highly heterogeneous model the ability of ACT to impact on transmission via gametocytocidal effects is also reduced because those that are treated are most likely to be in the highly exposed group who are quickly reinfected (Figure Va, 5 lowest transmission settings and Vb, 2 lowest transmission settings). However prophylactic drug effects can have greater impact when there is high heterogeneity because more of the highly exposed group are protected and most infectious bites are received by them (Figure Va, highest transmission setting and Vb, four highest transmission settings). A lower degree of heterogeneity (20% of people receive 70% of mosquito bites) than that assumed in the main model appears less likely in some settings since the rate of infection is lower than the rate of clinical episodes.

**Figure V. Sensitivity analysis: effect of varying heterogeneity of exposure on the main results: reductions in rates of clinical episodes by a short-acting ACT and a long-acting non-gametocytocidal drug across the six transmission settings in Tanzania. All other parameters are kept constant. The legend shows % of human population in high exposure group : % mosquito bites received by high exposure group. Scenarios where the rate of clinical incidence is higher than the rate of infection in that setting are assumed to be unrealistic and not shown. Results in other settings using same parameter combination are shown with unconnected lines to indicate the parameters may not be valid.**

(a)

(b)

short-acting ACT

long-acting non-gametocytocidal drug

The assumed properties of ACT are also important (Figure VI). In the lowest transmission setting, the percentage reductions in clinical episodes ranged from 38-58% using minimum and maximum estimates of impact on gametocytaemia, and if prophylactic time was also varied, the range in percentage reduction was 36-66%. In the highest transmission setting, the range of impact was greater: clinical episodes were reduced by between 7% and 49% when effects on gametocytaemia were varied, and by 2-71% when prophylactic time was also varied. Looking in more detail at effects of treatment on gametocytaemia shows that reducing the duration of infection generally has more impact on transmission than does reducing infectiousness by the same factor (results not shown). However in our two lowest transmission settings or when the relative reduction is small, a given percentage reduction in either duration of infection or infectiousness produces comparable impact.

Overall even relatively small variations in impact on gametocytaemia are important. Clinical trials measuring patient infectiousness show that the number of doses of artemisinin derivative have an important influence: a 6-dose regimen of artemether lumefantrine (AL) had significantly higher impact on gametocytaemia than the 3-dose regimens sulphadoxine-pyrimethamine-artesunate (SP-AS) and chloroquine-artesunate (CQ-AS) [10]. In our main model we use a combined best estimate of ACT impact on infectiousness obtained from pooled data on patients who were treated with 3-dose SP-AS or 6-dose AL. The majority of patients were in the SP-AS group (n=174) rather than the AL group (n=75) [46] so our best estimate is reasonably conservative. Other antimalarials with greater impact on gametocytaemia could substantially raise the impact of treatment. The prophylactic time of the partner drug in a chosen ACT combination also has significant implications for transmission reductions, particularly at high transmission.

**Figure VI. Sensitivity analysis: effects of varying ACT impact size on reductions in clinical episodes across the six Tanzanian transmission settings.**

The results are relatively robust to the remaining parameters explored. The parameters shown in figures VIIa-d (proportion of symptomatic cases which are treated and duration of symptoms) both ultimately affect the proportion of treatment received by symptomatic individuals and thus individuals at the more highly infectious early stage of their infection. However because total treatment rates in the population are kept constant to match survey treatment data, the effect of decreasing treatment among symptomatics is to increase the amount of treatment which is received by asymptomatic individuals in any other state regardless of whether they are infected. Interestingly this does not make a large difference to transmission impact, although it does make some difference to the impact of an ACT programme combined with improved diagnostic testing (Figure VIII). If more treatment is assumed to be taken by those without symptomatic malaria, fewer treatments are in those with any patent infection and so introducing improved diagnosis reduces treatment rates further and therefore ACT impact is lower. Figures VIIe-f show the model results are fairly robust to variation in the duration of the infectious period during its patent and subpatent phase except for ACT impact in the low transmission settings. Assuming a shorter duration of infection means the baseline infection incidence estimate is higher in order to reproduce the observed prevalence. Due to increased difficulty of reducing transmission at a higher initial incidence rate (Figure IV simplified model), impact is reduced. If a very long duration of infection is assumed, the estimated clinical incidence rate is often higher than the estimated incidence of infection. Changing the assumed size of pre-ACT non-artemisinin treatment impact on transmission at baseline has a small effect on the results estimates (Figure VIIg-h). Assuming higher pre-ACT impact means baseline infection incidence is estimated to be higher, which as before can reduce treatment impact on transmission (Figure IV simplified model). However ACT impact on the duration of infection is kept the same relative to the baseline non-artemisinin drug, so the absolute reduction in the infectious period can be greater than under the standard model estimates. Using population averages and disregarding age group variation in infectiousness, recovery times, force of infection and treatment rates has surprisingly little impact on the results, particularly in the three higher transmission settings (Figures VIIi-j). This is because the proportion of treatment received by each age group is relatively well in proportion to their contribution to the infectious reservoir (see equation for *L(i,t)* above) in these settings (results not shown).

**Figure VII. Sensitivity analysis of key parameters. Our main results were reproduced using low and high estimates of key parameters. Best estimate results from the main paper text are shown in light blue for short-acting ACT impact and red for the longer-acting non-gametocytocidal antimalarial. Scenarios where the rate of clinical incidence is higher than the rate of infection in that setting are assumed to be unrealistic and not shown. Results in other settings using same parameter combination are shown with unconnected lines to indicate the parameters may not be valid.**

**Figure VII (continued). Sensitivity analysis of key parameters.**

**Figure VIII. Sensitivity analysis of the impact of a programme introducing improved diagnostic testing together with ACT. The proportion of total treatments in the population received by symptomatic malaria cases in the pre-ACT scenario is varied by changing the probability of treatment of a symptomatic case (a) or the duration of symptoms (b). Scenarios where the rate of clinical incidence is higher than the rate of infection in that setting are assumed to be unrealistic and not shown. Results in other settings using same parameter combination are shown with unconnected lines to indicate the parameters may not be valid.**

(a)

(b)

**References**

1. Collins WE, Jeffery GM (2003) A retrospective examination of mosquito infection on humans infected with Plasmodium falciparum. Am J Trop Med Hyg 68: 366-371.

2. Aron JL, May RM (1982) The population dynamics of malaria. In: Anderson RA, editor. The population dynamics of infectious diseases: theory and applications. London: Chapman and Hall. pp. 139-179.

3. Dietz K, Molineaux L, Thomas A (1974) A malaria model tested in the African savannah. Bull World Health Organ 50: 347-357.

4. Jeffery GM, Eyles DE (1954) The duration in the human host of infections with a Panama strain of Plasmodium falciparum. Am J Trop Med Hyg 3: 219-224.

5. Babiker HA, Abdel-Muhsin AM, Ranford-Cartwright LC, Satti G, Walliker D (1998) Characteristics of Plasmodium falciparum parasites that survive the lengthy dry season in eastern Sudan where malaria transmission is markedly seasonal. Am J Trop Med Hyg 59: 582-590.

6. Missinou MA, Kun JF, Lell B, Kremsner PG (2001) Change in Plasmodium falciparum genotype during successive malaria episodes in Gabonese children. Parasitol Res 87: 1020-1023.

7. Mercereau-Puijalon O (1996) Revisiting host/parasite interactions: molecular analysis of parasites collected during longitudinal and cross-sectional surveys in humans. Parasite Immunol 18: 173-180.

8. Bruce MC, Day KP (2002) Cross-species regulation of malaria parasitaemia in the human host. Curr Opin Microbiol 5: 431-437.

9. Lusingu JP, Vestergaard LS, Mmbando BP, Drakeley CJ, Jones C, et al. (2004) Malaria morbidity and immunity among residents of villages with different Plasmodium falciparum transmission intensity in North-Eastern Tanzania. Malar J 3: 26.

10. Okell L, Drakeley C, Ghani AC, Bousema JT, Sutherland C (2008) Reduction of transmission from malaria patients by artemisinin combination therapies: a pooled analysis of six randomized trials. Malar J 7: 125.

11. Olliaro P, Pinoges L, Checchi F, Vaillant M, Guthmann JP (2008) Risk associated with asymptomatic parasitaemia occurring post-antimalarial treatment. Trop Med Int Health 13: 83-90.

12. National Bureau of Statistics of the United Republic of Tanzania (2003) Tanzania 2002 population and housing census.

13. Smith T, Charlwood JD, Takken W, Tanner M, Spiegelhalter DJ (1995) Mapping the densities of malaria vectors within a single village. Acta Trop 59: 1-18.

14. Woolhouse ME, Dye C, Etard JF, Smith T, Charlwood JD, et al. (1997) Heterogeneities in the transmission of infectious agents: implications for the design of control programs. Proc Natl Acad Sci U S A 94: 338-342.

15. Smith T, Maire N, Dietz K, Killeen GF, Vounatsou P, et al. (2006) Relationship between the entomologic inoculation rate and the force of infection for Plasmodium falciparum malaria. Am J Trop Med Hyg 75: 11-18.

16. Owusu-Agyei S, Koram KA, Baird JK, Utz GC, Binka FN, et al. (2001) Incidence of symptomatic and asymptomatic Plasmodium falciparum infection following curative therapy in adult residents of northern Ghana. Am J Trop Med Hyg 65: 197-203.

17. Baird JK, Owusu Agyei S, Utz GC, Koram K, Barcus MJ, et al. (2002) Seasonal malaria attack rates in infants and young children in northern Ghana. Am J Trop Med Hyg 66: 280-286.

18. Sama W, Owusu-Agyei S, Felger I, Dietz K, Smith T (2006) Age and seasonal variation in the transition rates and detectability of Plasmodium falciparum malaria. Parasitology 132: 13-21.

19. Bonnet S, Gouagna LC, Paul RE, Safeukui I, Meunier JY, et al. (2003) Estimation of malaria transmission from humans to mosquitoes in two neighbouring villages in south Cameroon: evaluation and comparison of several indices. Trans R Soc Trop Med Hyg 97: 53-59.

20. Githeko AK, Brandling-Bennett AD, Beier M, Atieli F, Owaga M, et al. (1992) The reservoir of Plasmodium falciparum malaria in a holoendemic area of western Kenya. Trans R Soc Trop Med Hyg 86: 355-358.

21. Struik SS, Riley EM (2004) Does malaria suffer from lack of memory? Immunol Rev 201: 268-290.

22. Hay SI, Rogers DJ, Toomer JF, Snow RW (2000) Annual Plasmodium falciparum entomological inoculation rates (EIR) across Africa: literature survey, Internet access and review. Trans R Soc Trop Med Hyg 94: 113-127.

23. Gillies MT (1953) The duration of the gonotrophic cycle in Anopheles gambiae and Anopheles funestus, with a note on the efficiency of hand catching. East Afr Med J 30: 129-135.

24. Bruce-Chwatt (1960) A study of the blood-feeding patterns of Anopheles mosquitos through precipitin tests. Bull World Health Organ 22: 685-720.

25. Rickman LS, Jones TR, Long GW, Paparello S, Schneider I, et al. (1990) Plasmodium falciparum-infected Anopheles stephensi inconsistently transmit malaria to humans. Am J Trop Med Hyg 43: 441-445.

26. Beier JC, Oster CN, Onyango FK, Bales JD, Sherwood JA, et al. (1994) Plasmodium falciparum incidence relative to entomologic inoculation rates at a site proposed for testing malaria vaccines in western Kenya. Am J Trop Med Hyg 50: 529-536.

27. Macdonald G (1957) The epidemiology and control of malaria. London: Oxford University Press.

28. Garrett-Jones C, Shidrawi GR (1969) Malaria vectorial capacity of a population of Anopheles gambiae: an exercise in epidemiological entomology. Bull World Health Organ 40: 531-545.

29. Gillies MT (1961) Studies on the dispersion and survival of Anopheles gambiae Giles in East Africa, by means of marking and release experiments. Bull Entomological Res 52: 99-127.

30. Eyles DE, Young MD (1951) The duration of untreated or inadequately treated Plasmodium falciparum infections in the human host. J Natl Malar Soc 10: 327-336.

31. Nedelman J (1989) Gametocytaemia and infectiousness in falciparum malaria: observations and models. Adv Dis Vector Res 6: 59-89.

32. Falk N, Maire N, Sama W, Owusu-Agyei S, Smith T, et al. (2006) Comparison of PCR-rflp and genescan-based genotyping for analyzing infection dynamics of Plasmodium falciparum. Am J Trop Med Hyg 74: 944-950.

33. Sama W, Dietz K, Smith T (2006) Distribution of survival times of deliberate Plasmodium falciparum infections in tertiary syphilis patients. Trans R Soc Trop Med Hyg.

34. Bekessy A, Molineaux L, Storey J (1976) Estimation of incidence and recovery rates of Plasmodium falciparum parasitaemia from longitudinal data. Bull World Health Organ 54: 685-693.

35. Boudin C, Olivier M, Molez JF, Chiron JP, Ambroise-Thomas P (1993) High human malarial infectivity to laboratory-bred Anopheles gambiae in a village in Burkina Faso. Am J Trop Med Hyg 48: 700-706.

36. Miller MJ (1958) Observations on the natural history of malaria in the semi-resistant West African. Trans R Soc Trop Med Hyg 52: 152-168.

37. Bruce-Chwatt LJ (1963) A Longitudinal Survey of Natural Malaria Infection in a Group of West African Adults. I. West Afr Med J 12: 141-173.

38. Ruebush TK, Kern MK, Campbell CC, Oloo AJ (1995) Self-treatment of malaria in a rural area of western Kenya. Bull World Health Organ 73: 229-236.

39. Espino F, Manderson L (2000) Treatment seeking for malaria in Morong, Bataan, the Philippines. Soc Sci Med 50: 1309-1316.

40. Pearce RJ, Drakeley C, Chandramohan D, Mosha F, Roper C (2003) Molecular determination of point mutation haplotypes in the dihydrofolate reductase and dihydropteroate synthase of Plasmodium falciparum in three districts of northern Tanzania. Antimicrob Agents Chemother 47: 1347-1354.

41. Tarning J, Ashley EA, Lindegardh N, Stepniewska K, Phaiphun L, et al. (2008) Population Pharmacokinetics of Piperaquine after Two Different Treatment Regimens with Dihydroartemisinin-Piperaquine in Patients with Plasmodium falciparum Malaria in Thailand. Antimicrob Agents Chemother 52: 1052-1061.

42. Watkins WM, Mberu EK, Winstanley PA, Plowe CV (1997) The efficacy of antifolate antimalarial combinations in Africa: a predictive model based on pharmacodynamic and pharmacokinetic analyses. Parasitol Today 13: 459-464.

43. Ezzet F, van Vugt M, Nosten F, Looareesuwan S, White NJ (2000) Pharmacokinetics and pharmacodynamics of lumefantrine (benflumetol) in acute falciparum malaria. Antimicrob Agents Chemother 44: 697-704.

44. National Bureau of Statistics of the United Republic of Tanzania, USAID, ORC Macro (2005) Tanzania Demographic and Family Health Survey 2004-2005.

45. Chandler CI, Drakeley CJ, Reyburn H, Carneiro I (2006) The effect of altitude on parasite density case definitions for malaria in northeastern Tanzania. Trop Med Int Health 11: 1178-1184.

46. Bousema JT, Schneider P, Gouagna LC, Drakeley CJ, Tostmann A, et al. (2006) Moderate Effect of Artemisinin-Based Combination Therapy on Transmission of Plasmodium falciparum. J Infect Dis 193: 1151-1159.
